# Conformational Isomerization of Imide Anions Governs Solvation and Transport in Water-in-Salt Electrolytes

**DOI:** 10.1021/jacs.6c06055

**Published:** 2026-06-11

**Authors:** Huong T.D. Nguyen, Lingzhe Fang, Volodymyr Koverga, Lalita Rai, Mohammed Lemaalem, Xingyi Lyu, Anh T. Ngo, Tao Li

**Affiliations:** 1 Department of Chemistry, Virginia Tech, Blacksburg, Virginia 24061, United States; 2 Department of Chemistry and Biochemistry, 2848 Northern Illinois University, DeKalb, Illinois 60115, United States; 3 Department of Chemical Engineering, 14681 University of Illinois Chicago, Chicago, Illinois 60607, United States; 4 Material Science Division, Argonne National Laboratory, Lemont, Illinois 60439, United States; 5 Chemistry and Material Science Group, X-ray Science Division, Argonne National Laboratory, Lemont, Illinois 60439, United States

## Abstract

The behavior of highly concentrated electrolytes departs radically from the dilute-solution theory, yet the molecular origin of this transformation remains unresolved. Here, we identify the conformational isomerization of molecular ions as a decisive, previously unrecognized control parameter governing structure and transport in crowded aqueous electrolytes. Across a series of fluorosulfonimide anions, we show that increasing concentration drives a collective shift from extended transoid to compact cisoid conformers, revealed by small-angle X-ray scattering, vibrational spectroscopy, pulsed-field gradient NMR, and molecular dynamics simulations. This conformational transition triggers a collapse of the hydrogen-bonded water network and the emergence of densely packed ionic domains with confined water, producing a qualitative change in Li^+^ transport from solvent-mediated diffusion to network-confined hopping. Anion size and asymmetry systematically tune the onset of this transition, demonstrating that molecular geometry dictates mesoscale organization and dynamics in the ion-rich regime. Our results establish ion conformation, not merely composition or coordination, as a fundamental thermodynamic variable in concentrated solutions, providing a chemical framework that unifies solvation structure and transport in water-in-salt electrolytes and suggesting new principles for designing dense ionic media.

## Graphical abstract

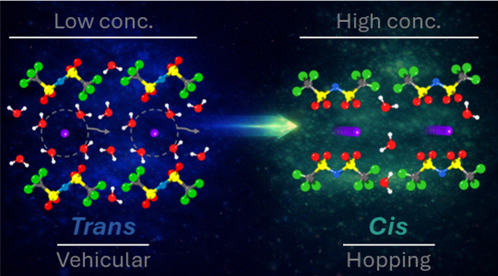

## Introduction

Rechargeable aqueous lithium-ion batteries (ALIBs) offer an appealing route toward safe and low-cost grid-scale energy storage, yet their energy density is fundamentally constrained by the ∼1.23 V stability window of dilute aqueous electrolytes.^1,2^ The advent of highly concentrated “water-in-salt” electrolytes (WISEs) alleviates this limitation by suppressing water activity and extending the electrochemical stability window to ∼ 3 V.^3–9^ Among them, imide-based lithium salts are particularly effective owing to their high solubility and electrochemical robustness.^10–12^ Previous studies combining vibrational spectroscopy,^13–15^ nuclear magnetic resonance (NMR),^16,17^ scattering techniques,^18–21^ and molecular dynamics (MD) simulations^22–25^ have established a general picture in which increasing salt concentration drives a progression from solvent-separated ion pairs to contact ion pairs and extended aggregates, defining the local hydration environment in WISEs. However, a key molecular variable remains largely unexplored: the intrinsic conformational degree of freedom of the imide anion. The transoid and cisoid geometries, distinguished by CF_3_ groups on the opposite or same side of the S–N–S plane, introduce subtle yet potentially decisive differences in charge distribution and steric accessibility.^26–28^ We hypothesize that this conformational bias governs solvation organization, mesoscale aggregation, and ultimately ion transport in concentrated aqueous electrolytes.^29–32^

Here, we combine small-angle X-ray scattering (SAXS), Raman and Fourier-transform infrared (FTIR) spectroscopy, pulsed-field-gradient NMR (PFG-NMR), and MD simulations to examine the conformational behavior of imide-based lithium salts in aqueous solutions. Because each technique provides different sensitivity to the nanoscale structure, conformational evolution, and transport dynamics, the methods were applied to complementary subsets of the electrolyte series rather than identically to every anion. SAXS was used across the full set of symmetric and asymmetric imide-based salts to compare concentration-dependent nanoscale organization. Vibrational spectroscopy was applied to representative systems (LiTFSI–TFSI and LiBETI–BETI) where conformational signatures are well resolved and can be reliably quantified, enabling direct observation of the concentration-driven transoid-to-cisoid transition. Meanwhile, PFG-NMR and MD simulations were focused primarily on BETI- and BNTI-based families to systematically examine the effects of fluorocarbon side-chain length and anion asymmetry on ion transport and nanoscale organization. Both symmetric anions (FSI–FSI^–^, TFSI–TFSI^–^, BETI–BETI^–^, and BNTI–BNTI^–^) and asymmetric analogues (BETI–FSI^–^, BETI–TFSI^–^, BNTI–FSI^–^, and BNTI–TFSI^–^) are investigated (Figure 1a). Across multiple systems, we identify a concentration-driven transition from the transoid to the cisoid conformer, which is not previously reported in aqueous electrolytes. Vibrational spectroscopy reveals a systematic increase in the cisoid population with increasing salt concentration, whereas MD simulations reproduce this trend and link it to changes in solvation and ionic clustering. Transport measurements further show that anion conformation directly governs Li^+^, anion, and water mobility. At high concentrations, cisoid and asymmetric anions enable tighter ionic packing and faster suppression of diffusion, accompanied by stronger ion–ion correlations and restricted Li^+^ motion. These results establish anion isomerization as a previously overlooked design parameter in water-in-salt electrolytes and provide a new handle for engineering high-energy, intrinsically safe aqueous batteries.

**Figure 1:**
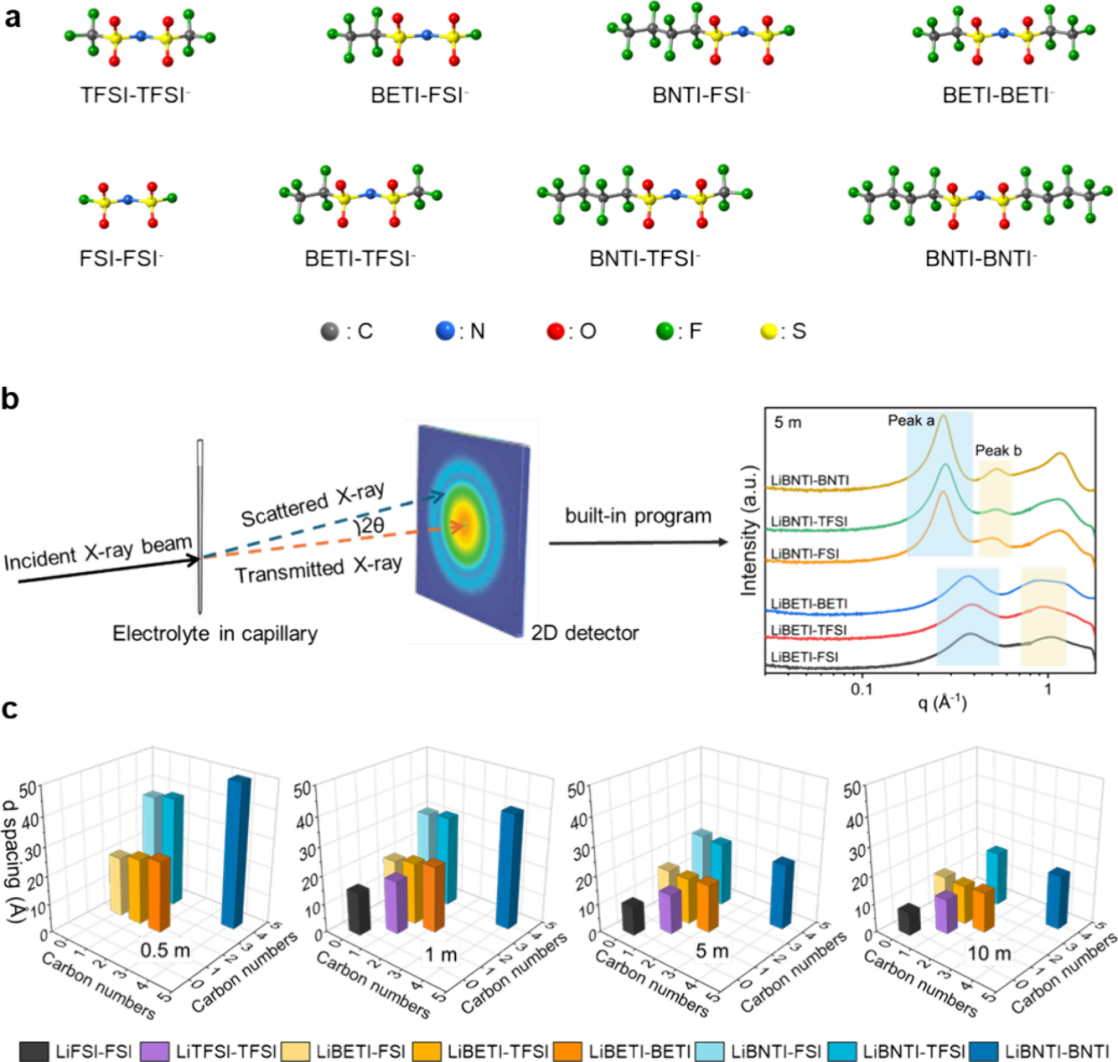
Concentration-dependent nanoscale organization of imide-based anions in aqueous electrolytes. (a) Molecular structures of imide-based anions: bis­(fluorosulfonyl)­imide (FSI–FSI^–^), bis­(trifluoromethylsulfonyl)­imide (TFSI–TFSI^–^), bis­(pentafluoroethanesulfonyl)­imide (BETI–BETI^–^), bis­(nonafluorobutanesulfonyl)­imide (BNTI–BNTI^–^), (fluorosulfonyl) (pentafluoroethanesulfonyl)­imide (BETI–FSI^–^), (trifluoromethanesulfonyl) (pentafluoroethanesulfonyl)­imide (BETI–TFSI^–^), (fluoro sulfonyl) (nonafluorobutanesulfonyl)­imide (BNTI–FSI^–^), and (trifluoromethanesulfonyl) (nonafluorobutane sulfonyl)­imide (BNTI–TFSI^–^). (b) Schematic of the experimental setup and representative SAXS profiles of imide-based Li salts at a concentration of 5 m. (c) Concentration-dependent evolution of the *d*-spacing corresponding to peak a as a function of carbon chain length on both sides of the anion.

## Results and Discussion

To probe how molecular structure governs nanoscale organization in WISEs, we performed SAXS on a series of symmetric and asymmetric imide-based lithium salts over a broad concentration range (0.5–20 m) (Figure 1b and Figures S1 and S2). Across all systems, the SAXS profiles exhibit two characteristic features associated with interanion correlations, reflecting the organization of solvated imide species in aqueous environments. The lower-*q* feature (peak a) arises from intermediate-range interdomain correlations, associated with the emergence of nanoscale heterogeneity and anion-rich domains, whereas the higher-*q* feature (peak b) reflects short-range ion–ion correlations, corresponding to local packing between neighboring anions and their solvation shells.^18,19,22,33–35^ As salt concentration increases, the dominant low-*q* feature systematically shifts to higher scattering vectors, indicating the progressive compression of interaggregate distances and a transition from loosely solvated anion clusters to denser, ion-rich networks. While the absolute *d*-spacing at low concentration scales with anion size, an unexpected convergence of nanoscale spacing emerges at high concentrations for chemically distinct anions (Figure 1c). This loss of size dependence suggests that factors beyond steric volume, specifically, concentration-induced conformational adaptation, govern nanoscale packing in the highly concentrated regime. The interpretation is further supported by the consistency between SAXS, Raman, FTIR, and MD results, which together provide a coherent multiscale picture linking molecular conformation, nanoscale organization, and transport behavior.

### Concentration-Dependent Structural Reorganization Governed by Anion Geometry

To better understand this behavior, we replotted the *d*-spacing values as a function of imide anion size, quantified by the total number of carbon atoms (*n*) in the fluorinated side chains. As shown in Figure 2b, comparisons between asymmetric LiBETI–FSI and symmetric LiTFSI–TFSI (*n* = 2), as well as between asymmetric LiBNTI–FSI and symmetric LiBETI–BETI (*n* = 4), reveal substantial differences in *d*-spacing despite identical carbon numbers. Notably, the *d*-spacing of LiBNTI–FSI exceeds that of LiBETI–BETI by more than ∼5 Å across the investigated concentration range (Figure 2c), and LiBETI–FSI consistently exhibits larger spacing than LiTFSI–TFSI. These results demonstrate that nanoscale organization is governed by factors beyond anion size alone. Strikingly, the *d*-spacing values of LiBETI–FSI and LiBETI–BETI converge at 5–10 m, indicating a concentration-driven structural transformation that diminishes the influence of chain length.

**Figure 2:**
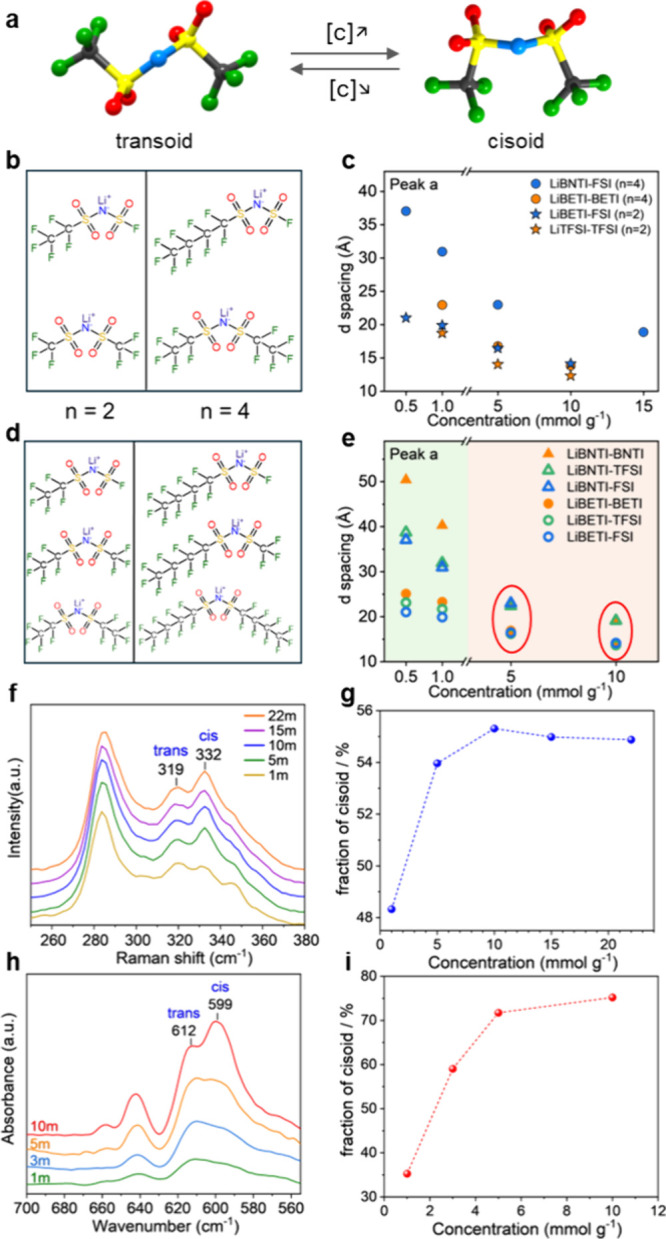
Integrated structural, SAXS, and spectroscopic analyses reveal a concentration-driven anion conformational evolution. (a) Schematic illustration of the concentration-driven transoid and cisoid conformation. (b) Molecular structures of symmetric and asymmetric imide-based salts, including LiBETI–FSI and LiTFSI–TFSI, with a total carbon number of 2 (*n* = 2), and LiBNTI–FSI and LiBETI–BETI, with *n* = 4. (c, e) Concentration-dependent variation of the *d*-spacing corresponding to peak a extracted from SAXS profiles. (d) Molecular structures of LiBETI–*X* (*X* = FSI, TFSI, BETI; left) and LiBNTI–*X* (*X* = FSI, TFSI, BNTI; right). (f) Raman spectra of LiTFSI–TFSI and (h) FTIR spectra of LiBETI–BETI aqueous solutions at different concentrations. (g, i) Fraction of cisoid and transoid conformers as a function of salt concentration for LiTFSI–TFSI and LiBETI–BETI, demonstrating a progressive shift from the transoid to the cisoid conformer with increasing concentration.

To probe this effect further, we fixed the longer fluorocarbon substituent of asymmetric anions while systematically varying the shorter chain (Figure 2d,e). At low concentrations (0.5–1 m), the *d*-spacing of peak a increases monotonically with carbon number, consistent with steric expansion. At higher concentrations,^5–10^ however, the *d*-spacing converges across all salts independent of chain length. We interpret this convergence as being consistent with the concentration-driven conformational adaptation of the imide anions (Figure 2a), together with the enhanced ion–ion correlations and reduced free volume at high concentration. The extended transoid geometry, favored in dilute, solvent-rich environments, becomes unfavorable as free volume decreases, while the more compact cisoid conformation enables more efficient packing at high ionic strength.^26,29,36,37^ This interpretation is consistent with solid-state crystallographic studies by Xue et al.,^38^ which showed that TFSI^–^ can adopt both transoid and cisoid geometries depending on coordination and hydration. In lithium salts, the cisoid conformer is preferentially stabilized and promotes layered packing with fluorinated domain segregation-structural motifs that closely mirror the concentration-induced nanoscale organization observed in our SAXS measurements.

### Conformational Transition Indicated by Raman and FTIR Spectroscopy

To directly probe the proposed trans–cis conformational transition, we employed vibrational spectroscopy, which is highly sensitive to the geometry of the sulfonimide backbone.^39–46^ Raman spectra of LiTFSI–TFSI aqueous electrolytes show a clear concentration-dependent redistribution of conformers (Figure 2f). At low concentrations,^1^ the spectrum is dominated by a band at 319 cm^–1^, characteristic of the transoid conformer favored in solvent-rich environments. With increasing concentration,^5–20^ the intensity of the 332 cm^–1^ band, assigned to the cisoid conformer, increases systematically (Figure 2g and Figure S3), indicating a progressive shift in conformational equilibrium driven by ionic crowding and reduced free volume. Quantitative analysis shows that the cisoid population increases from approximately 48% at low concentration to 55% at high concentration, demonstrating that conformational isomerization evolves continuously with ionic strength and directly correlates with the emergence of densely packed ionic environments. FTIR spectroscopy of LiBETI–BETI (Figure 2h) independently confirms this trend. The bands at 612 and 599 cm^–1^ correspond to the transoid and cisoid conformers, respectively.^44–47^ At low concentrations, the transoid band dominates, whereas higher salt content leads to a pronounced growth of the cisoid feature. Quantitative peak fitting reveals that the cisoid population increases from approximately 35% at low concentrations to 75% at high concentrations (Figure 2i and Figure S4), consistent with the enhanced ionic packing and reduced free volume at elevated salt concentrations. Together, the Raman and FTIR results provide direct spectroscopic evidence for a concentration-driven trans-to-cis transition, linking local anion geometry to the SAXS-observed convergence of nanoscale packing and establishing conformational adaptation as a key structural mechanism in imide-based WISEs.

### Molecular Dynamics (MD) Simulations Reveal Concentration-Driven Structural and Transport Evolution

Building on the conformational trends identified by Raman and FTIR spectroscopy for representative TFSI- and BETI-based systems, we employed classical molecular dynamics (MD) simulations to obtain a comprehensive molecular-level picture of structure and dynamics across the full set of imide-based electrolytes. The conformational analysis was focused on the BETI- and BNTI-based systems because these anions provide a chemically consistent series for separating the effects of side-chain length and asymmetry. As shown in Figure 3c and Figure S5, the simulated structure factors closely reproduce the experimental SAXS profiles, capturing peak positions and *d*-spacings with an average mean absolute deviation of ∼7.8%, validating the reliability of the simulations. At low salt concentrations, structural correlations are dominated by water–anion interactions (Figure 3d and Figures S6–S8), consistent with solvent-separated ions and conformationally flexible anions stabilized in transoid geometries. With increasing concentration (Figure 3e), the SAXS correlations sharpen and shift to higher *q*, indicating reduced intermolecular distances and the emergence of more compact ionic organization. This transition reflects a crowding-driven reorganization from solvent-separated anion clusters to densely packed ion networks, accompanied by the progressive confinement of water within ion-rich domains. In parallel, Li^+^ solvation evolves from predominantly water-coordinated environments (A0W5/A0W6; Figure S9) toward mixed water–anion coordination (A1W4/A2W3), signaling enhanced ion association and reduced free volume.

**Figure 3:**
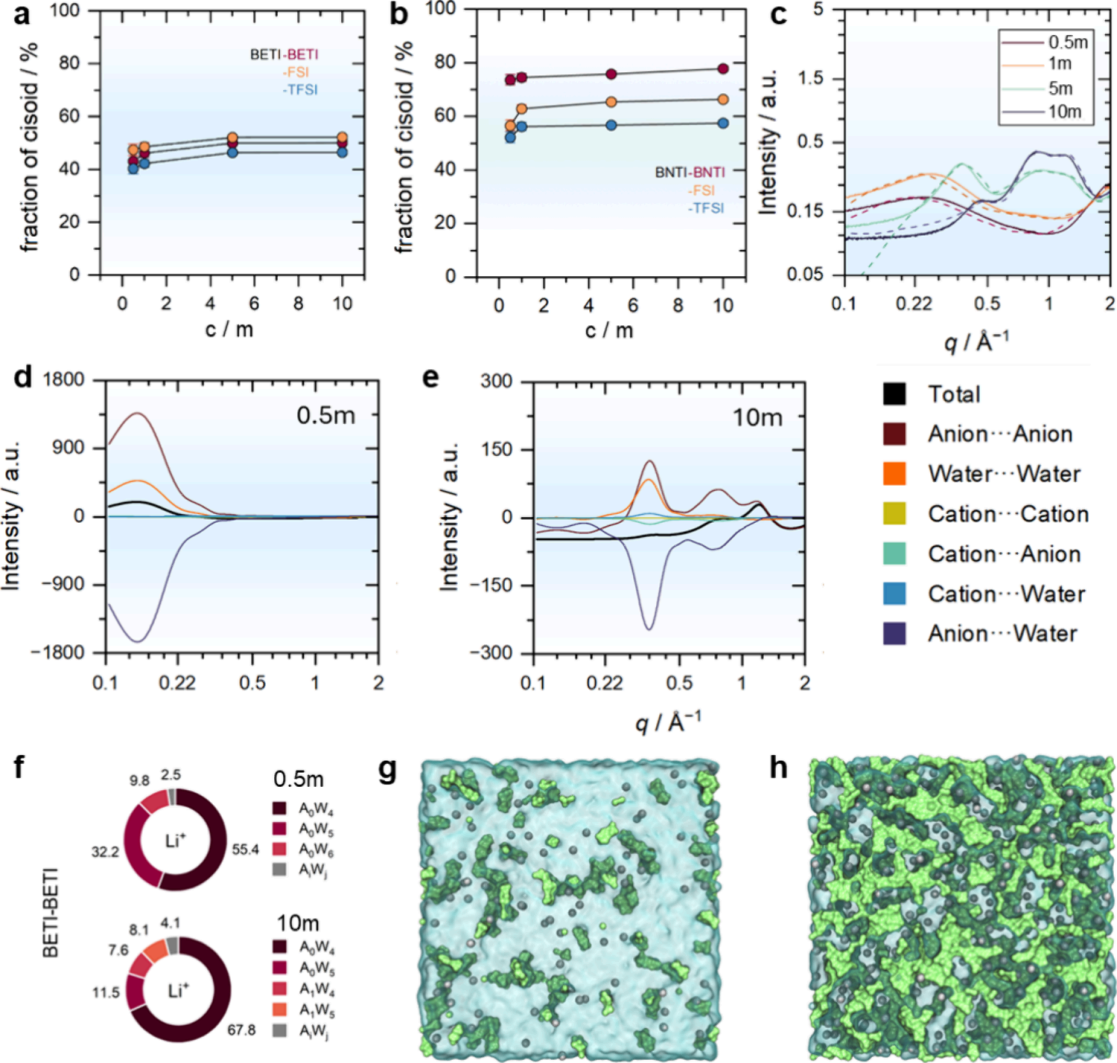
MD analyses reveal a concentration-driven reorganization of imide-based aqueous electrolytes. (a, b) Fraction of the cisoid conformer as a function of salt concentration for BETI- and BNTI-based systems obtained from MD simulations. (c) Experimental SAXS profiles compared with simulated structure factors (dashed lines) for 0.5, 1, 5, and 10 m LiBETI–BETI aqueous electrolytes. (d, e) Decomposition of the SAXS profiles for 0.5 and 10 m LiBETI–BETI aqueous electrolytes. (f) Illustration of lithium cation (Li^+^) solvation environment statistics expressed as the mutual probability of anion (A) and water (W) configurations (A_*i*_W_*j*_) to be in the proximity to Li^+^ for 0.5 and 10 m LiBETI–BETI aqueous electrolyte. The A_*i*_W_*j*_ configurations with lower occurrence <7% were gathered in separate a gray block. (g, h) Representative MD snapshots of 1 and 5 m LiBETI–BETI aqueous electrolytes illustrating the transition from discrete anion clusters to extended ionic networks accompanied by reduced void space (green: anion-rich domains; blue: water; gray spheres: Li^+^).

These structural changes directly stabilize compact anion conformations. Quantification of cisoid–transoid populations (Figure 3a,b) reveals a monotonic increase in the cisoid fraction with salt concentration across all systems, marking a shift from solvent-stabilized, flexible transoid geometries at low concentrations to compact cisoid conformers favored under ionic crowding and stronger ion–ion interactions. BNTI-based electrolytes consistently exhibit higher cisoid populations than BETI analogues, indicating that increased steric bulk enhances the stabilization of compact conformations, while FSI- and TFSI-containing systems show more rapid cisoid enrichment due to the earlier development of direct ion contact. Importantly, the rise in cisoid population tracks the sharpening of SAXS correlation features, establishing a direct link between nanoscale packing, reduced molecular flexibility, and anion conformational locking in the ion-rich regime.

To quantitatively connect anion conformation with ion transport, we combined PFG-NMR measurements (Figure 4c,d and Figure S18) with MD simulations. Both approaches reveal consistent concentration-dependent trends across all electrolytes. At low salt concentrations, PFG-NMR and MD show that all systems preserve the mobility hierarchy H_2_O > Li^+^ > anion, reflecting a continuous hydrogen-bonded water network, preferential Li^+^ hydration, and conformationally flexible anions dominated by transoid geometries. In this regime, anions in BETI-based electrolytes exhibit higher diffusivities than BNTI analogues, consistent with weaker steric constraints and reduced hydrophobic segregation that sustain more fluid solvation environments. As salt concentration increases, both experiments and simulations show a sharp decline in diffusivities for all species, coinciding with the breakdown of water connectivity, increased anion participation in Li^+^ solvation, and growth of ion clusters. Systems with faster cisoid enrichment (particularly those containing asymmetric anions) show a more pronounced reduction in diffusivity, whereas symmetric anions display a more gradual decline. To elucidate the microscopic origin of this behavior, MD-resolved van Hove correlation functions were analyzed. At low concentrations (Figure 4e), Li^+^ displacement distributions are broad, indicative of solvent-mediated (vehicular) diffusion (Figure 4a) enabled by flexible anion conformations and transient ion associations. At high concentration (Figure 4f,b), the distributions narrow markedly, signaling the strong localization of Li^+^ within ion-rich domains. This localization correlates directly with the concentration-driven increase in cisoid anion populations: compact cisoid conformers restrict rotational freedom, strengthen persistent Li^+^–anion coordination, and promote efficient ionic packing, thereby suppressing long-range motion. Notably, asymmetric anions facilitate more efficient ionic packing than symmetric analogues, leading to a more rapid decline in diffusivity (Figure 4c,d inset).

**Figure 4:**
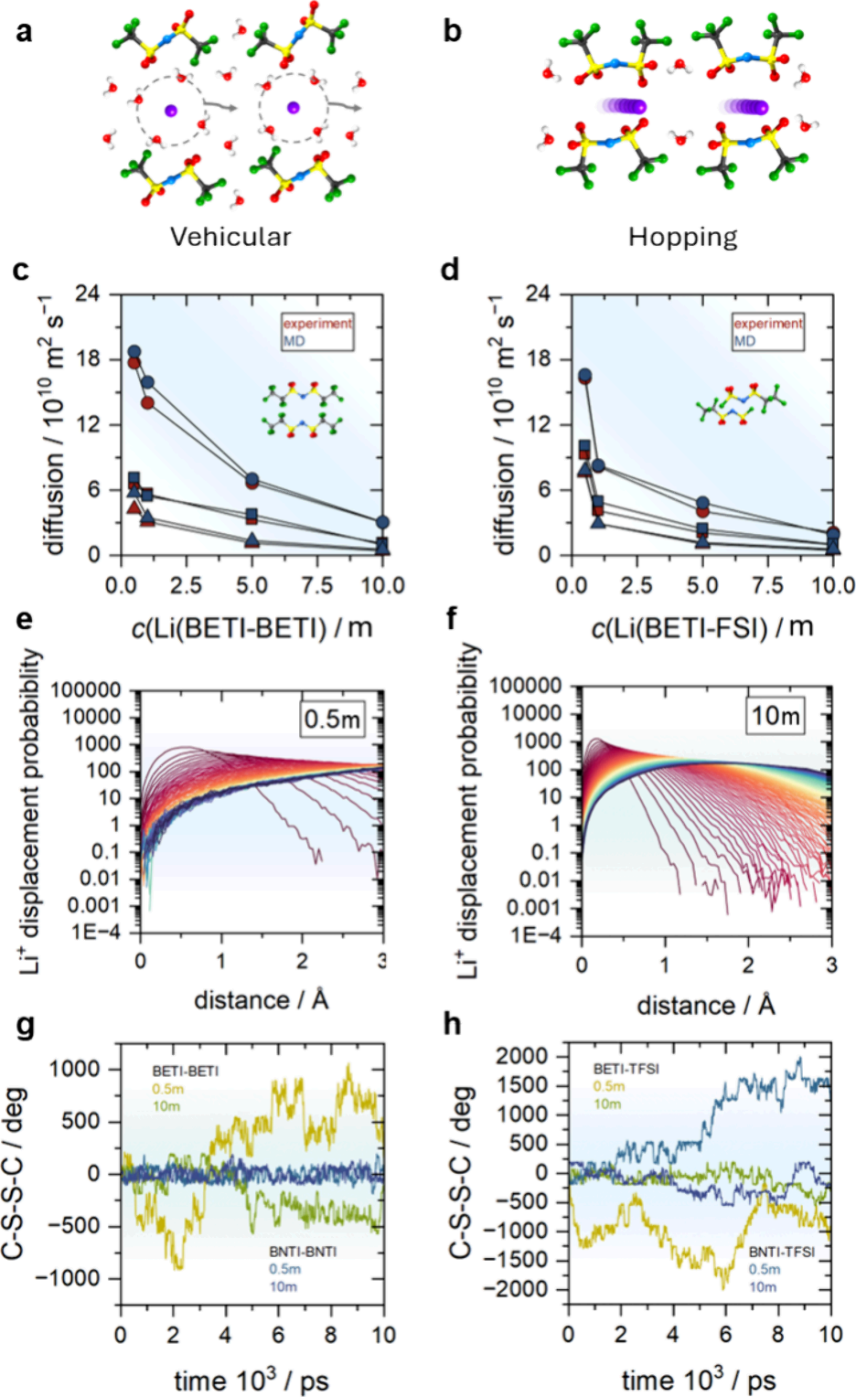
Influence of anion conformation on ion transport in imide-based aqueous electrolytes. Schematic illustration of Li^+^ transport mechanisms: (a) solvent-mediated (vehicular) diffusion at low concentration and (b) cisoid facilitated, network confined hopping at high concentration. (c, d) Experimental and MD derived self-diffusion coefficients of Li^+^ (squares), anions (triangles), and H_2_O (circles) as a function of concentration for BETI- and BNTI-based systems; inset: representative anion–anion arrangements of cisoid conformers at high concentration for symmetric (BETI–BETI) and asymmetric (BETI–FSI) anion. (e, f) Van Hove probability distributions of Li^+^ displacements at low (0.5 m) and high (10 m) concentrations in LiBETI–BETI aqueous electrolyte showing a transition from vehicular to hopping mechanism. The color gradient represents the evolution from short-time to long-time displacement probability distributions. (g, h) Time evolution of the C–S–S–C dihedral angle describing the relative orientation of the two fluorinated sulfonyl side chains and used to distinguish transoid and cisoid conformations. Full rotations were included in the temporal evolution, resulting in continuous angular traces.

Consistently, the dihedral angle dynamics (Figure 4g,h) shows pronounced suppression of anion rotational freedom at high salt content, indicating conformational locking in the ion-dense regime. Together, the agreement between PFG-NMR and MD establishes a concentration-driven crossover from solvent-mediated Li^+^ transport to network-confined hopping, governed by anion cis–trans isomerization. Anion conformational locking thus emerges as the key molecular mechanism linking nanoscale structure to macroscopic transport in WISEs.

## Conclusions

In summary, we reveal a concentration-driven trans-to-cis conformational transition of imide-based anions in aqueous WISEs and show that this previously overlooked degree of freedom governs both the nanoscale organization and Li^+^ transport. At low concentrations, transoid conformers dominate within a continuous water network, enabling solvent-mediated Li^+^ diffusion. Increasing the salt content stabilizes compact cisoid conformers through steric confinement and enhanced ion association, promoting dense ionic networks and a crossover to network-confined Li^+^ hopping. The onset and extent of this transition are modulated by anion size and symmetry: BETI-based electrolytes preserve water connectivity and higher diffusivities, whereas BNTI-based systems undergo more rapid structural collapse and mobility suppression. Within the same BETI- or BNTI-based framework, asymmetric anions facilitate more efficient ionic packing than symmetric analogues, resulting in a faster reduction in the diffusivity. These results establish anion conformational adaptability as a key structural parameter linking the solvation architecture to collective ion dynamics, providing new molecular design principles for high-performance aqueous lithium-ion batteries.

## Experimental Section

### Sample Preparation

Lithium bis­(trifluoromethanesulfonyl)­imide (LiTFSI–TFSI, >99%, Sigma-Aldrich), lithium bis­(pentafluoroethanesulfonyl)­imide (LiBETI–BETI, >99%, TCI), and lithium bis­(nonafluorobutanesulfonyl)­imide (LiBNTI–BNTI, >95%, Provisco CS) were used as symmetric salts. Asymmetric salts including lithium (fluorosulfonyl)­(pentafluoroethanesulfonyl)­imide (LiBETI-FSI, >95%, Provisco CS), lithium (trifluoromethanesulfonyl)­(pentafluoroethanesulfonyl)­imide (LiBETI-TFSI, >95%, Provisco CS), lithium (fluorosulfonyl)­(nonafluorobutanesulfonyl)­imide (LiBNTI-FSI, >95%, Provisco CS), and lithium (trifluoromethanesulfonyl)­(nonafluorobutanesulfonyl)­imide (LiBNTI-TFSI, >95%, Provisco CS) were also used. All salts were dissolved in high-purity water (resistivity 18.2 MΩ·cm at 25 °C). Electrolyte solutions were prepared at varying concentrations, defined by molality (mol kg^–1^ of solvent), and are reported as abbreviated values of 0.5, 1, 5, 10, 15, and 20 m.

### Small-Angle X-ray Scattering (SAXS)

SAXS measurements were conducted at beamline 12-ID-B of the Advanced Photon Source (APS) at Argonne National Laboratory. Scattering data were collected using a Pilatus 2M detector (DECTRIS Ltd.) with an incident X-ray energy of 13.3 keV. The two-dimensional (2D) scattering patterns were radially integrated to obtain one-dimensional intensity profiles, *I*(*q*), as a function of the scattering vector *q*, where *q* = 4π sin θ/λ. Calibration of the *q*-scale was performed using silver behenate as a standard. Electrolyte samples were loaded into 1.5 mm diameter quartz capillaries and hermetically sealed with epoxy to prevent evaporation during measurements. (Figures S1 and S2)

### Fourier Transform Infrared (FTIR) Spectroscopy

FTIR measurements were carried out using a Shimadzu IRAffinity-1S spectrometer equipped with a diamond attenuated total reflectance (ATR) accessory. Spectra were collected at room temperature under ambient conditions, and each sample was measured without further preparation (Figure S3).

### Raman Spectroscopy

Raman measurements were carried out at the Center for Nanoscale Materials (CNM), Argonne National Laboratory, using a Renishaw inVia Raman microscope with a 532 nm laser source. Samples were loaded into glass capillaries (1.5 mm inner diameter) and sealed with epoxy prior to the analysis (Figure S4).

### Pulsed Field Gradient Nuclear Magnetic Resonance (PFG-NMR)

NMR measurements were performed on a 500 MHz Bruker Avance III 500 spectrometer. Coaxial cells were used for the NMR measurements, and D_2_O was used as a reference. Self-diffusion coefficients of Li^+^, anions, and H_2_O were determined by ^7^Li, ^19^F, and ^1^H PFG-NMR, respectively, at 298 K. PFG-NMR measurements were conducted using a 5 mm BBFO probe equipped with *z*-axis gradient coils capable of producing gradients up to 50 G/cm. The *ledbpgp2s* pulse sequence (longitudinal eddy-current delay bipolar gradient pulse pair with two spoil gradients) was applied to minimize eddy-current effects and suppress convection. For each resonance, the gradient strength (*g*) was incremented linearly over 16–32 steps from 0 to 50 G/cm. The gradient pulse duration (*δ*) was set to 1.8 ms and the diffusion delay (Δ) to 99.9 ms, with a 5 ms longitudinal eddy-current delay inserted between bipolar gradient pulses. Sixteen scans were accumulated for each gradient increment using a recycle delay of 1 ms. The normalized signal attenuation, *S*(*g*)/*S*_0_, was analyzed using the Bruker Dynamics Center software and fitted to the Stejskal–Tanner equation:^48^

$$S(g)=S_{0}e^{-D\gamma^{2}g^{2}\delta^{2}(\Delta -\frac{\delta }{3})}$$

where *S­(g)* represents the observed integral, *S*_0_ is the zero-gradient integral, *D* is the diffusion coefficient (m^2^/s), *γ* is the gyromagnetic ratio of the observed nucleus (1.654 × 10^3^ Hz/G for ^7^Li, 4.006 × 10^3^ Hz/G for ^19^F, and 4.258 × 10^3^ Hz/G for ^1^H), *g* is the gradient strength (G/cm), *δ* is the gradient pulse duration (ms), and *Δ* is the diffusion time (ms). Nonlinear least-squares fitting was used to extract diffusion coefficients (Figures S12–S17).

### Computational Details

For classical molecular dynamics (MD) simulation, seven aqueous electrolyte systems involving Li­(BETI–BETI), Li­(BETI–FSI), Li­(BETI–TFSI), Li­(BNTI–BNTI), Li­(BNTI–FSI), Li­(BNTI–TFSI), and Li­(TFSI–TFSI) were selected. For water potentials a rigid type nonpolarizable SPC/E, “Simple Point Charge-Extended” model^49^ was used and for the potential parameters for anions^50^ were taken from the CL&P force field^51–55^ expressed by OPLS, “Optimized Potentials for Liquid Simulations”, analytical form for potential energy^56^ to adjust the intra- and interatomic potential parameters, describing covalent terms within bond stretching, angle bending and dihedral angle torsion along the covalent bond, and noncovalent terms represented by the van der Waals and Coulomb interactions:

$$U(r,\theta ,\varphi )=\sum_{\mathrm{bond}}k_{r}\Delta r^{2}+\sum_{\mathrm{angle}}k_{\theta }\Delta \theta^{2}+\sum_{\mathrm{dihedral}}\sum_{n=1,5}A_{n}\cos^{n-1}(\varphi )$$

where *U* is the sum over the internal terms as a function of atomic coordinates represented by bond distances (*r*), angles (θ), and dihedrals (φ); the parameters *k*_*r*,θ_ and *A*_*n*_ are the respective force constants and the variables. Although the use of a nonpolarizable force field may affect the absolute values of ion diffusivities and local coordination strengths, the CL&P-based parameters combined with the rigid SPC/E water model are expected to provide a reliable description of the concentration-dependent trends across the related fluorosulfonimide anions.

To generate input files for MD simulations, all potential parameters were adapted to the input-file syntax of the Moltemplate code.^57^ The initial coordinates of aqueous electrolytes containing the numbers of structural units corresponding to experimental concentrations of 0.5, 1, 5, and 10 m were generated using the Packmol code.^58^ All the structural units corresponding to the considered system were randomly placed in orthorhombic boxes with three-dimensional periodic boundary conditions. The box size was set to 85 Å. The distance tolerance between atoms belonging to different structural units was set to 2.0 Å to avoid unphysically close contacts in the initial configurations.

All the MD simulations presented in this work were conducted using the LAMMPS (“Large-scale Atomic/Molecular Massively Parallel Simulator) code v080223.^59^ The equations of motion were integrated with a time step of 1 fs. This time step was chosen to ensure stable integration of the flexible anions, while the geometry of water molecules was constrained using the SHAKE algorithm.^60^ Intramolecular nonbonded interactions followed the OPLS formalism, with 1–2 and 1–3 interactions excluded and 1–4 Lennard–Jones and Coulomb interactions scaled by a factor of 0.5. The electrostatic long-range interactions were evaluated using the computationally efficient Particle-particle particle-mesh method with an accuracy of 10^–5^, while a cutoff of 12 Å was used for the real-space Coulomb contribution and Lennard–Jones interactions. Each system was first minimized using the steepest descent algorithm with the default convergence criterion. Next, a 5 ns equilibration run was performed in the isothermal–isobaric (NpT) ensemble to adjust the system density and remove residual stresses associated with the initial packing. The system densities were further validated with experimental ones (Table S1). The final equilibrated configurations were then used as starting points for the production simulations conducted in the canonical ensemble (NVT) for 10 ns to collect the coordinates for subsequent structural and dynamical analysis. The temperature and pressure were maintained at 298 K and 1 atm using the Nosé–Hoover thermostat and barostat^61–63^ with coupling constants of 300 and 800 fs, respectively.

The trajectory from the production stage was saved every 1 ps, resulting in a total of 10,000 frames considered for analysis. For structural properties, including radial distribution functions, solvation structure statistics, conformational organization analysis, and structure factor analysis, every 10th frame along the trajectory was used, resulting in a total of 1000 analyzed frames. For the partial structure-factor analysis, the simulated systems were partitioned into three molecular/ionic atom groups: imide anions, water molecules, and Li ions. Within each group and their cross-combinations, all atoms were included in the analysis. Intramolecular and intermolecular contributions were not separated in the partial decomposition. For dynamical properties, including self-diffusion coefficients, the self-part of the van Hove correlation function, and conformational dynamics, the full set of saved frames was used. The self-diffusion coefficients were estimated from the mean-squared displacement of the centers of mass of the respective structural units with a correlation depth corresponding to 30.33% of the total trajectory length. All analyses discussed in this work were performed using the TRAVIS (“TRajectory Analyzer and VISualizer”) code version 062922,^64,65^ except for the conformational–organization analysis, which was carried out using an in-house Python code.

## Supplementary Material



## Notes

SAXS profiles of all electrolytes; Raman peak fitting for LiTFSI–TFSI; FTIR peak fitting for LiBETI–BETI; additional MD simulation results; and PFG-NMR diffusion analyses for BETI- and BNTI-based systems (PDF)

## Notes

All codes used for simulation and analysis are available from the corresponding authors upon request.
