# Supplementary material for: Conformational Isomerization of Imide Anions Governs Solvation and Transport in Water-in-Salt Electrolytes
Source: J Am Chem Soc. 2026 Jun 11;148(29):31011–9. doi: 10.1021/jacs.6c06055 (PMC13426250; doi:10.1021/jacs.6c06055)
Supplement: Supplementary file 1 [file ja6c06055_si_001.pdf]

Supporting Information for

Conformational Isomerization of Imide Anions Governs Solvation and Transport in Water-in-Salt Electrolytes

Huong T. D. Nguyen[1,‡], Lingzhe Fang[2,‡], Volodymyr Koverga[3,4,‡], Lalita Rai[2], Mohammed Lemaalem[3], Xingyi Lyu[2], Anh T. Ngo[3,4,], and Tao Li[1,5*]*

[1]Department of Chemistry, Virginia Tech, Blacksburg, Virginia 24061, United States

[2]Department of Chemistry and Biochemistry, Northern Illinois University, DeKalb, Illinois 60115, United States

[3]Department of Chemical Engineering, University of Illinois Chicago, Chicago, Illinois 60607, United States

[4]Material Science Division, Argonne National Laboratory, Lemont, Illinois 60439, United States

[5]Chemistry and Material Science Group, X-ray Science Division, Argonne National Laboratory, Lemont, Illinois 60439, United States

1. SAXS analysis

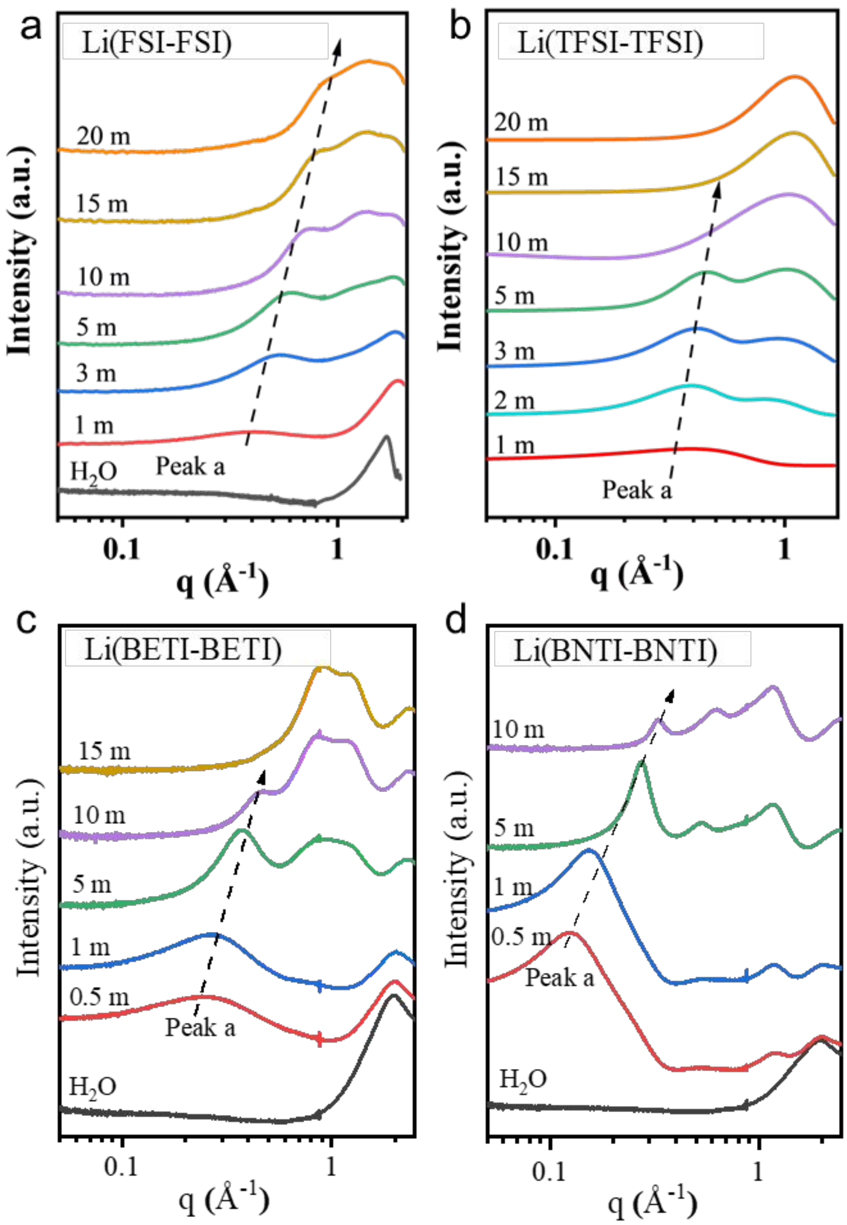

Figure S1. SAXS profiles of asymmetric imide-based lithium salts in aqueous solutions at various concentrations and ambient temperature: (a) lithium bis(fluorosulfonyl)imide (LiFSI-FSI), (b) lithium (fluorosulfonyl) (pentafluoroethanesulfonyl)imide (LiBETI-FSI), (c) lithium bis(pentafluoroethanesulfonyl)imide (LiBETI-BETI), (d) lithium bis(nonafluorobutanesulfonyl)imide (LiBNTI-BNTI).

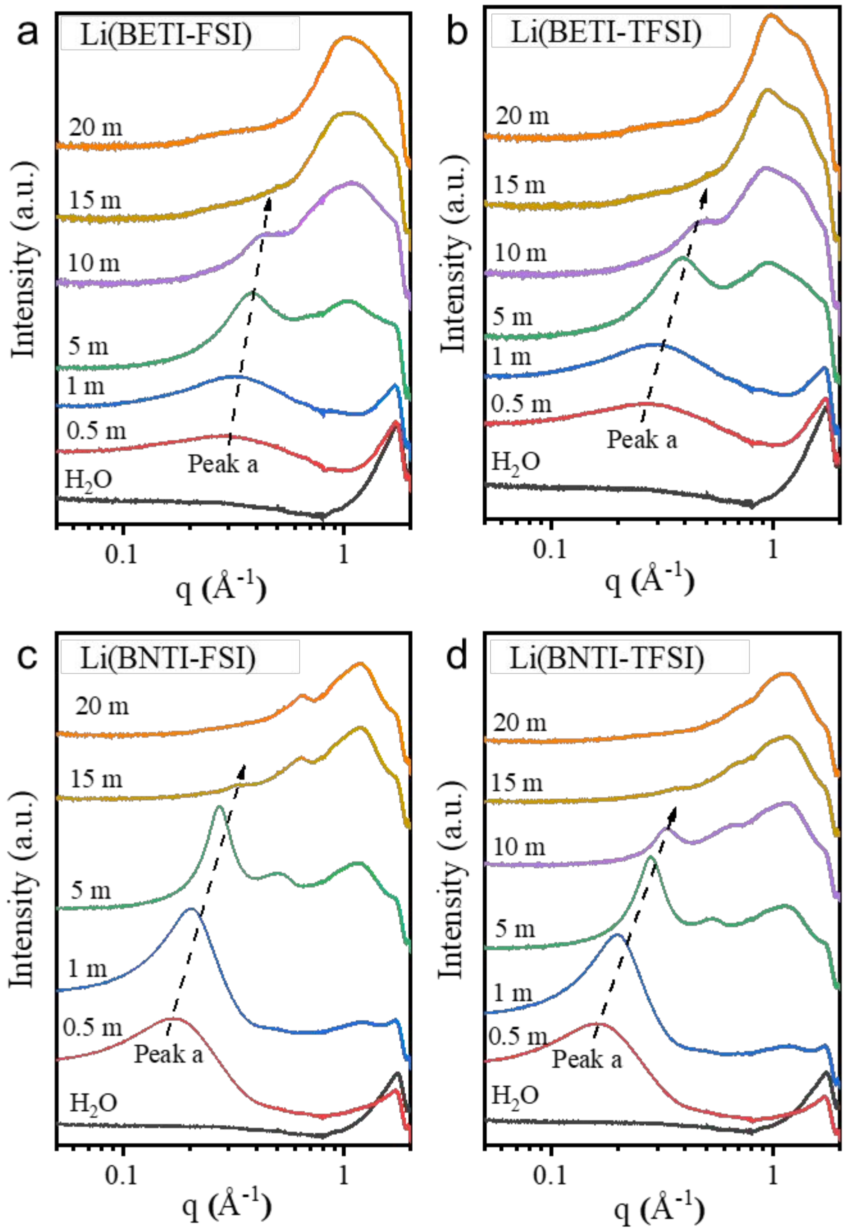

Figure S2. SAXS profiles of asymmetric imide-based lithium salts in aqueous solutions at various concentrations and ambient temperature: (a) lithium (fluorosulfonyl) (pentafluoroethanesulfonyl)imide (LiBETI-FSI), (b) lithium (trifluoromethanesulfonyl) (pentafluoroethanesulfonyl)imide (LiBETI-TFSI), (c) Lithium (fluoro sulfonyl) (nonafluorobutanesulfonyl)imide (LiBNTI-FSI), (d) Lithium (trifluoromethanesulfonyl) (nonafluorobutane sulfonyl)imide (LiBNTI-TFSI) aqueous solutions at different concentrations and ambient temperature.

2. Raman fitting curves

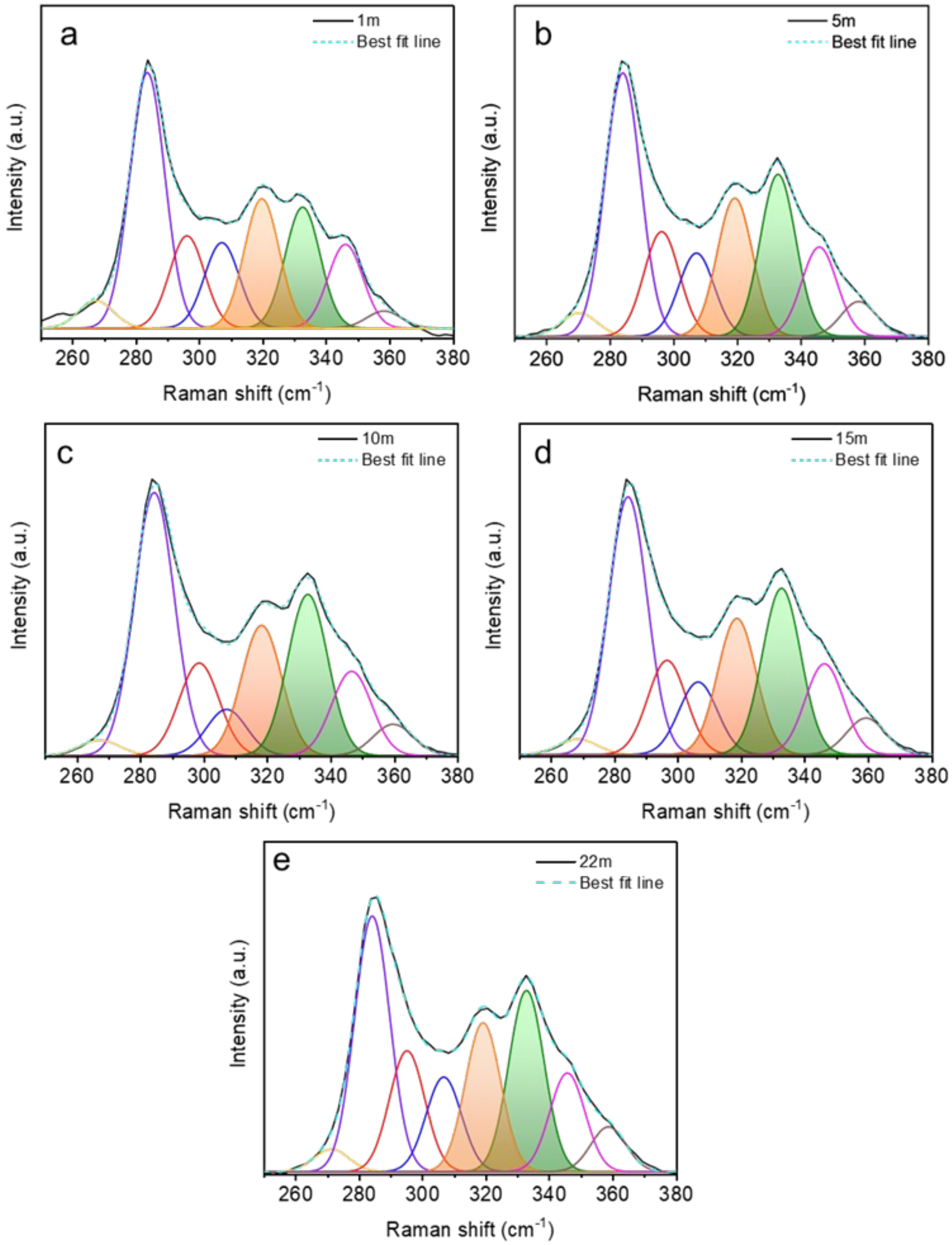

Figure S3. Raman spectra and corresponding fitting curves of LiTFSI-TFSI aqueous solutions at various concentrations.

3. FT-IR fitting curves

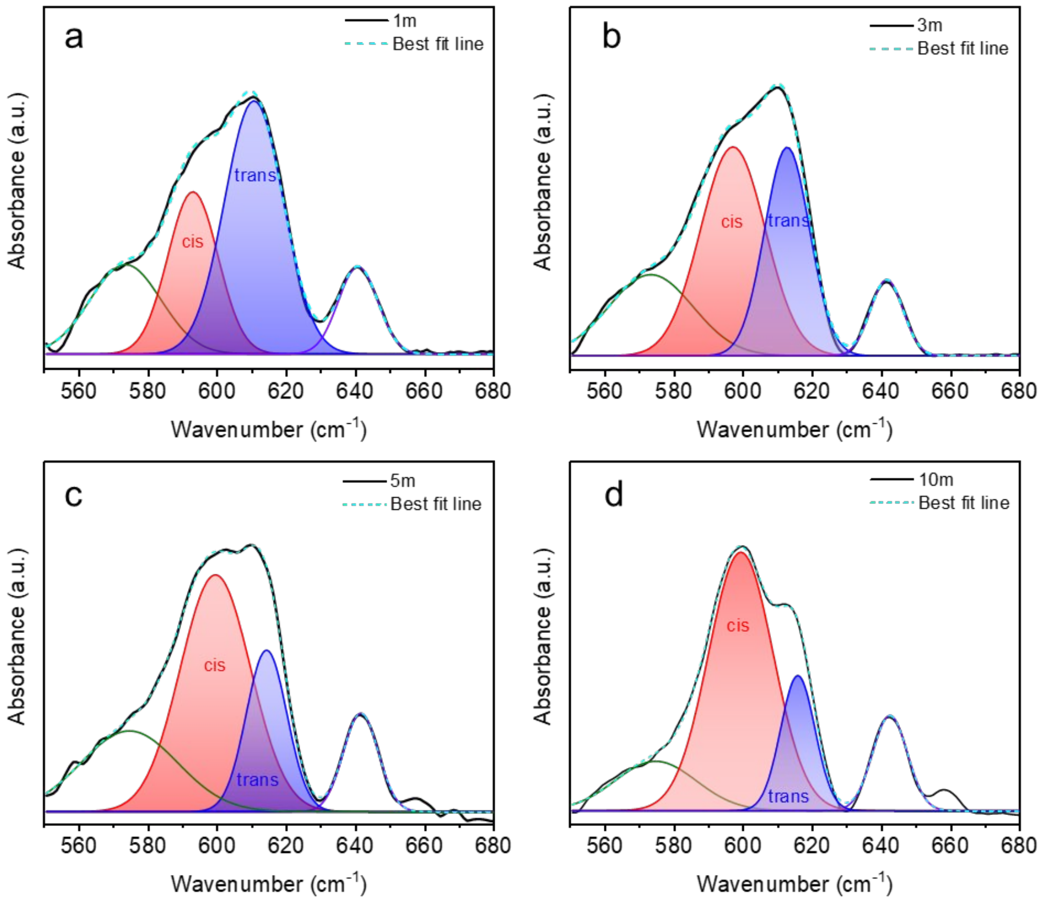

Figure S4. FT-IR spectra and corresponding fitting curves of LiBETI-BETI aqueous solutions at various concentrations.

4. MD simulation for SAXS analysis

Prior to the analysis of the underlying interactions, the obtained trajectories were validated based on their ability to reproduce experimental densities, as well as SAXS measurements. Density is considered as the key factor in the initial stages of MD simulation, since it determines the packing of the structural units (atoms or molecules) within the simulation box, and thus the local structural organization of the considered system. A higher density implies a more compact packing and, therefore, shorter average interatomic distances, and *vice versa*. This effect is also reflected in the resulting structure factor analysis, where higher packing and shorter interatomic distances shift the positions of peaks toward higher scattering vectors. Therefore, upon examining the calculated densities, they were found to be in good agreement with experimental measurements, with the mean absolute deviation not exceeding *ca* 0.1%, whereas the largest deviation of 4.83% was observed for the Li(BNTI-BNTI) system. Moreover, the increase in density with Li-salt concentration provides a general understanding of the behavior of the peaks in SAXS profiles.

Table S1. Aqueous electrolyte composition used for MD simulation and comparison simulated densities with experimental ones.

c / M	# salt	# water	density / g mL^{-1}	
			simulation	experiment
		BETI-BETI		
0.5	135	14901	1.08711	1.095
1	246	13602	1.16037	1.164
5	721	7991	1.47205	1.457
10	983	5444	1.62723	1.651
		BETI-FSI		
0.5	138	15325	1.06678	1.07637
1	259	14332	1.12682	1.13847
5	856	9482	1.41936	1.43778
10	1186	6571	1.58956	1.58933
		BETI-TFSI		
0.5	138	15325	1.07548	1.10257
1	258	14283	1.14094	1.17845
5	856	9482	1.43504	1.42469
10	1067	5911	1.59049	1.61115
		BNTI-BNTI		
0.5	124	13677	1.12494	1.08874
1	215	11885	1.22036	1.163
5	552	6117	1.5681	1.491
10	685	3793	1.71065	1.616

		BNTI-FSI		
0.5	136	15004	1.08852	1.10255
1	246	13634	1.16223	1.16677
5	695	7695	1.48875	1.49342
		BNTI-TFSI		
0.5	136	15004	1.09618	1.12558
1	247	13669	1.17441	1.21173
5	682	7555	1.49719	1.49342
10	904	5006	1.65103	1.67182
		TFSI-TFSI		
0.5	140	15452	1.06356	1.06895
1	257	14225	1.11287	1.13
5	849	9411	1.39183	1.427
10	1178	6524	1.54545	1.578

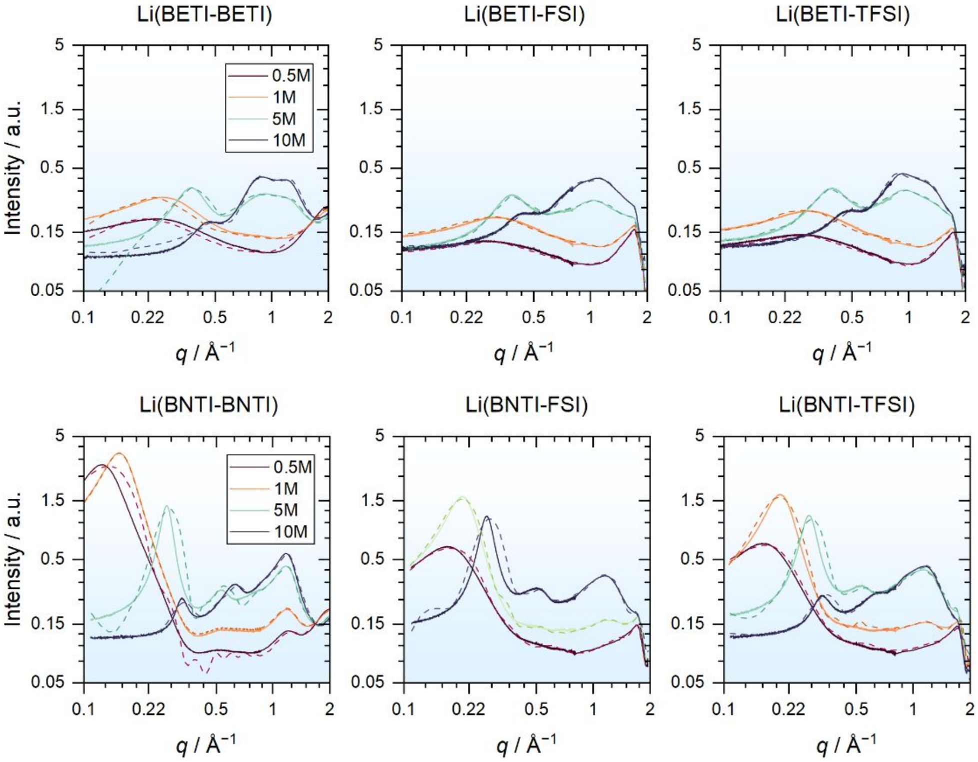

Figure S5. Illustration of experimental X-ray small angle scattering profiles in comparison with the structure factor obtained by means of molecular dynamics simulation of 0.5, 1, 5 and 10 m solutions of fluorosulfonimide-based Li-salt aqueous electrolytes

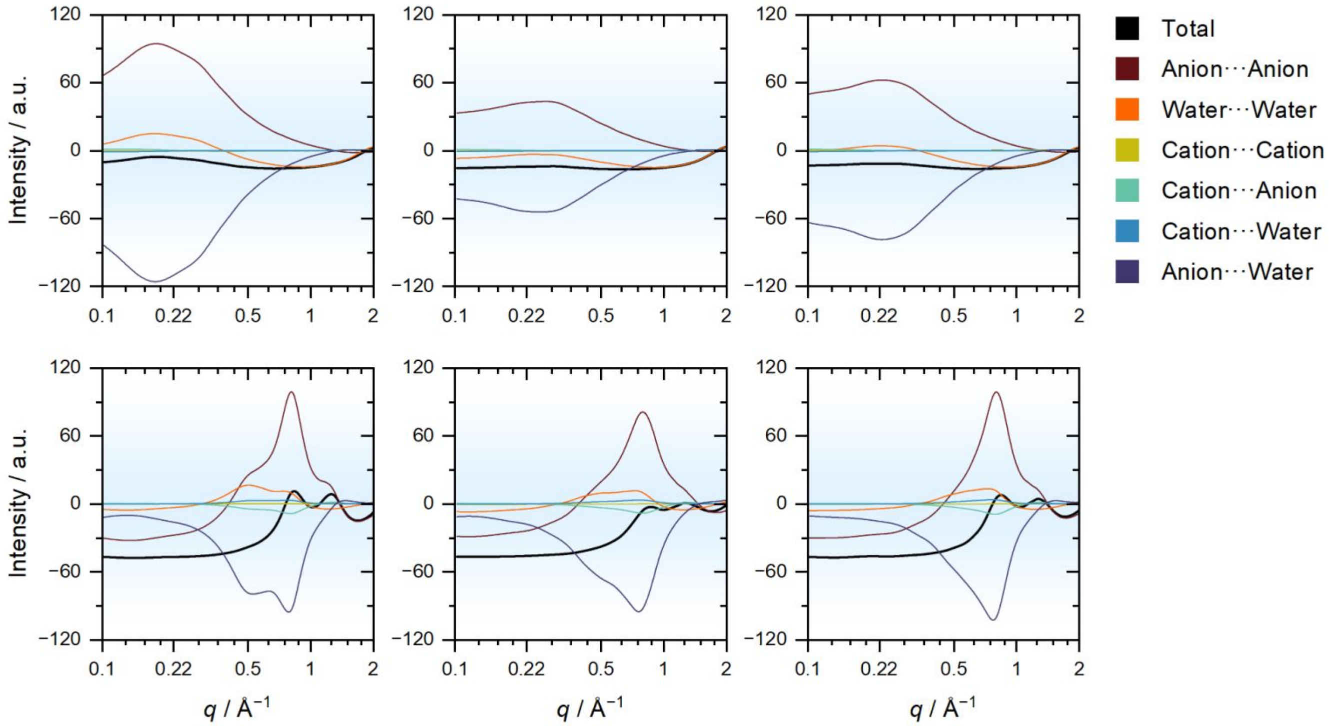

Figure S6. Illustration partial structure factor for BETI-based system: left – BETI-BETI, middle – BETI-FSI, right – BETI-TFSI for 0.5 m (top) and 10 m (bottom)

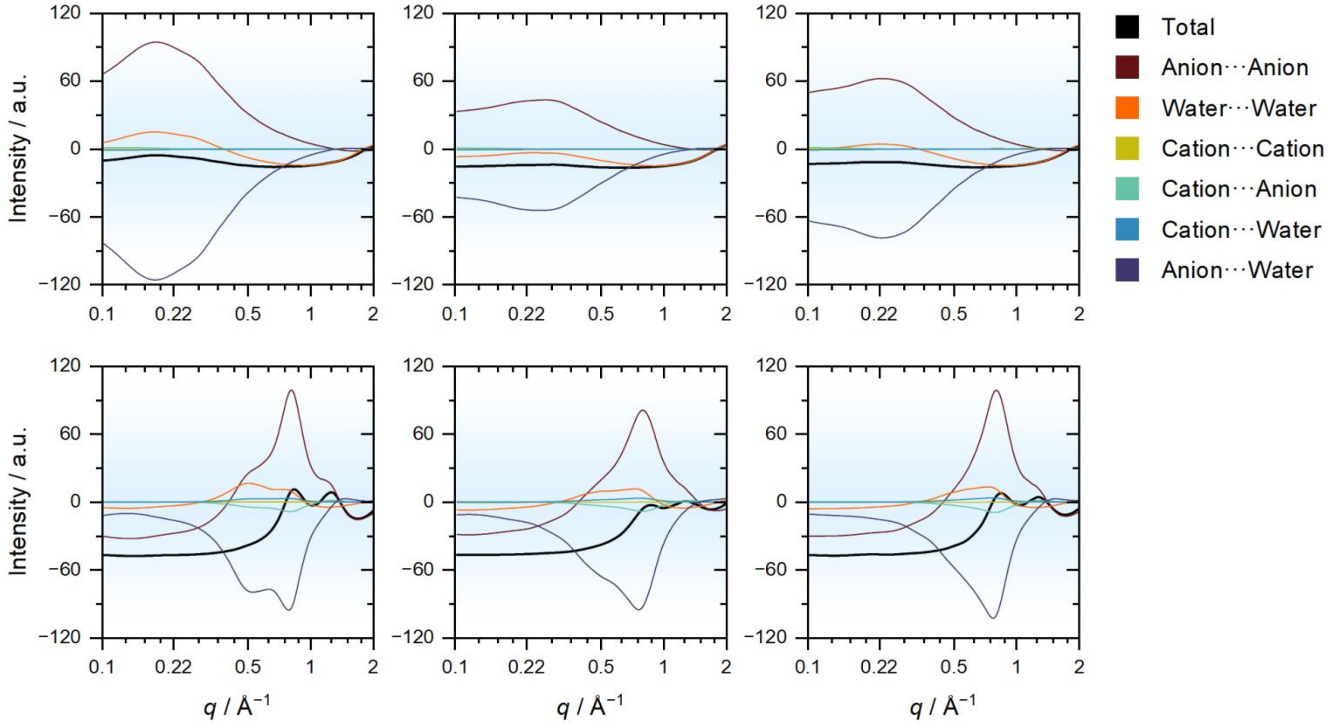

Figure S7. Illustration partial structure factor for BNTI-based system: left – BNTI-BNTI, middle – BNTI-FSI, right – BNTI-TFSI for 0.5 m (top) and 10 m (bottom)

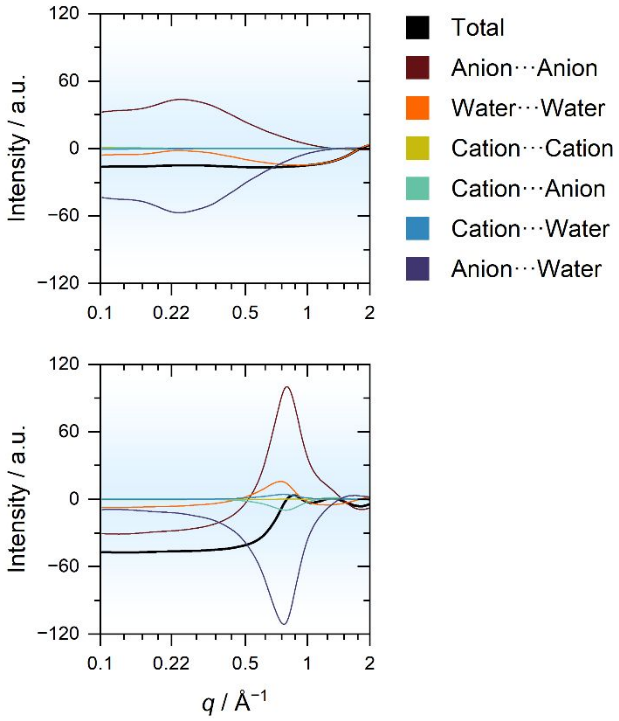

Figure S8. Illustration partial structure factor for LiTFSI-TFSI aqueous solution for 0.5 m (top) and 10 m (bottom)

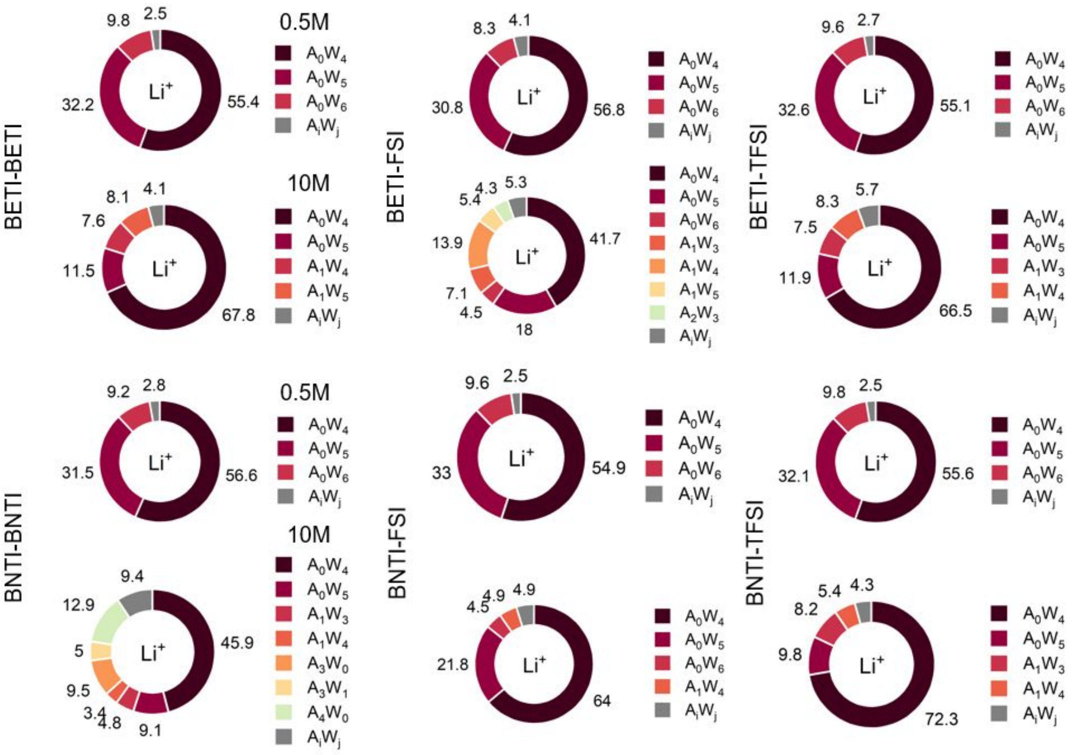

Figure S9. Illustration of lithium cation, Li$^+$, solvation environment statistics expressed as the mutual probability of anion, A, and water, W, configurations, A_iW_j, to be in the proximity to Li$^+$. The A_iW_j configurations with lower occurrence <7% were gathered in separate *grey* block

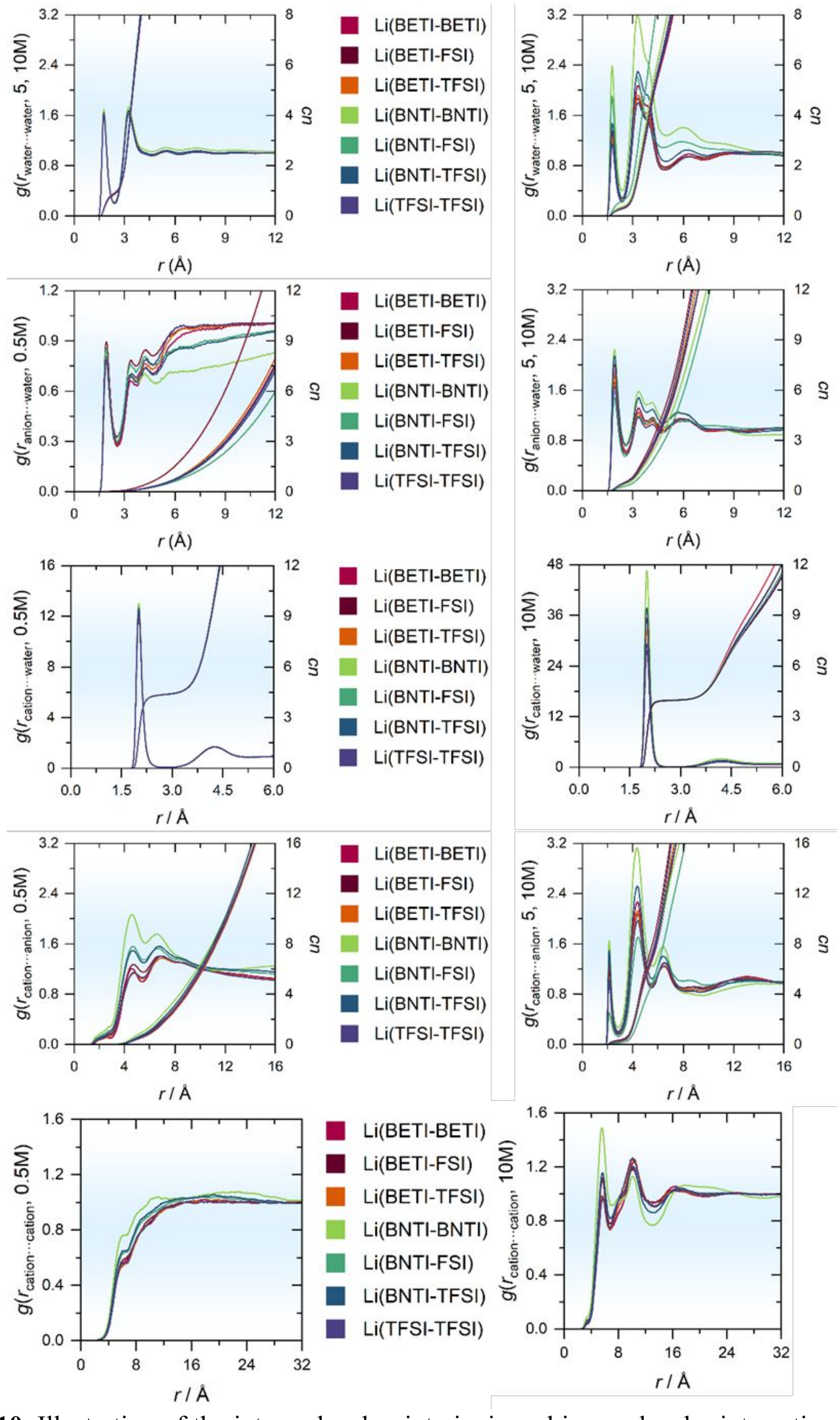

Figure S10. Illustration of the intermolecular, interionic and ion-molecular interactions between

the electrolyte constituents expressed by the representative pair radial distribution function, $g(r)$, between the positive *reference*, Li or H, and the most electronegative *observed*, O, water···water, cation···water, anion···water atomic sites in 0.5 and 10 m solutions (except for Li(BNTI-FSI) for which 5M was considered as the highest concentration) of fluorosulfonimide-based Li-salt aqueous electrolytes, where for anion···anion and cation···cation interactions, the Li and N atoms were used as the representative sites. The *right-hand-side* axis represents the coordination ability of the reference atom toward the observed one and expressed as the running coordination number, *cn*.

Radial distribution and coordination analyses reveal a concentration-driven reorganization of intermolecular and interionic interactions in fluorosulfonimide-based water-in-salt electrolytes **(Figure S10)**. At low salt concentrations, the water–water hydrogen-bond network dominates, with well-defined first and second solvation shells and minimal perturbation from ions, while anion–water and cation–anion interactions remain weak and dispersed. Increasing salt concentration progressively disrupts water hydrogen bonding, reduces water–water coordination by up to ~60%, and promotes stronger anion–water hydrogen bonding and enhanced cation–anion association, leading to ion pairing and cluster formation. BNTI-based systems exhibit stronger structuring effects on water and more pronounced ion–ion correlations than BETI- and TFSI-based electrolytes, reflecting steric and hydrophobic contributions. Overall, the interaction hierarchy evolves from water-dominated solvation to ion network-dominated organization, where stable Li^+–water coordination coexists with growing ionic aggregates embedded within increasingly heterogeneous water domains. Moreover, the anion size and symmetry play a central role in modulating intermolecular and interionic interactions in WISEs. Symmetric anions generally perturb water–water interactions less than asymmetric analogs, with BNTI-based anions showing weaker sensitivity to water structuring than BETI-based systems, while lower symmetry anions such as BETI-FSI and BNTI-FSI induce stronger disruption at high concentrations. Cation–water interactions are weakest in electrolytes containing the least symmetric anions, particularly in BETI-based systems, whereas higher anion symmetry enhances Li^+–water coordination. Interionic interactions follow a complementary trend, with cation–anion correlations weakest for BETI-based electrolytes, intermediate for TFSI systems, and strongest for BNTI-based salts, reflecting increasing steric bulk and hydrophobicity. As salt concentration increases, both cation–cation and anion–anion separations decrease, with anion–anion distances exhibiting a strong dependence on

anion chemistry and symmetry, highlighting the emergence of ion clustering and composition dependent nanoscale ordering.

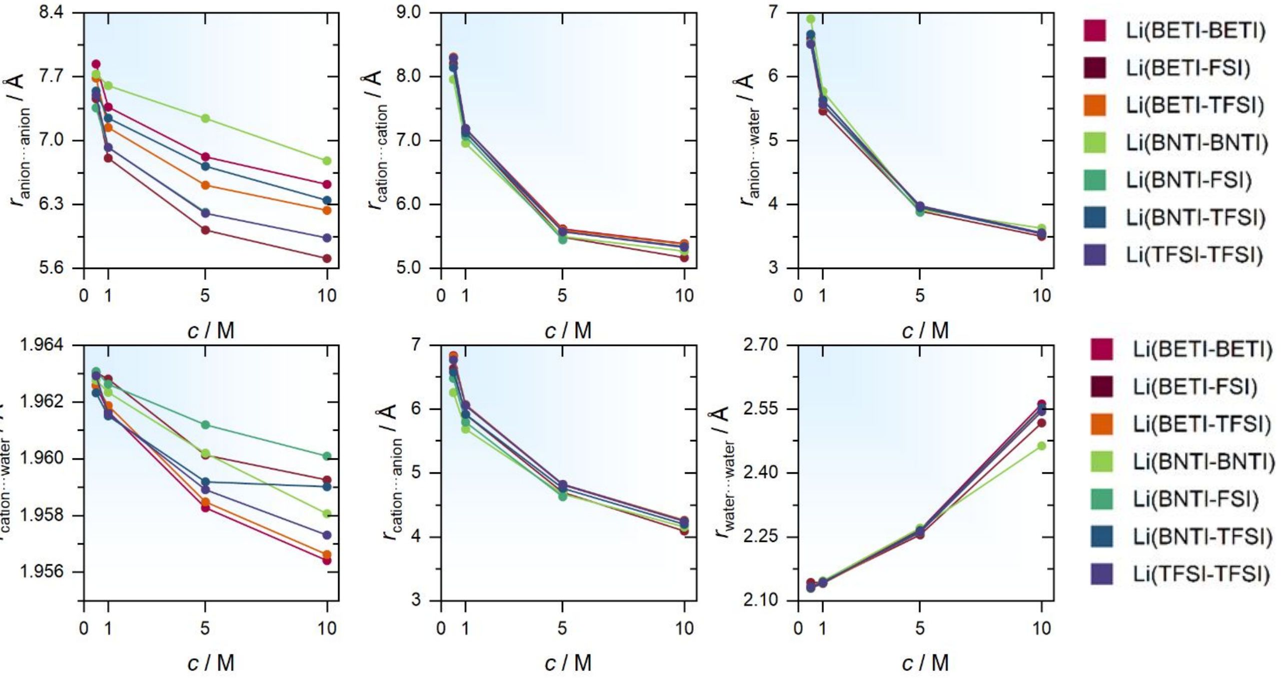

Figure S11. *Concentration-dependent average intermolecular distances obtained from MD simulations for different interaction pairs in aqueous Li imide-based salt electrolytes: anion–anion ($r_{anion-anion}$), cation–cation ($r_{cation-cation}$), anion–water ($r_{anion-water}$), cation–water ($r_{cation-water}$), cation–anion ($r_{cation-anion}$), and water–water ($r_{water-water}$) interactions as a function of salt concentration. The results compare symmetric and asymmetric imide-based systems, including Li(BETI-BETI), Li(BETI-FSI), Li(BETI-TFSI), Li(BNTI-BNTI), Li(BNTI-FSI), Li(BNTI-TFSI), and Li(TFSI-TFSI).*

Based on the evolution of average intermolecular distances **(Figure S11)**, at low salt concentrations the dominance of water molecules effectively shield Li^+ from extensive ion pairing, preserving a stable hydrogen bonding network among water molecules, as also reflected by the strong water–water correlations in the partial structure factor. In this regime, pronounced water–anion correlations indicate a water-rich solvation environment and the prevalence of water-coordinated Li^+ complexes. With increasing salt concentration, cation–anion and anion–water distances decrease, accompanied by sharpening and shifting of structure factor features to higher scattering vectors, signaling enhanced ionic ordering and clustering. This structural transition reflects the displacement of water molecules from Li^+ solvation shells by anions and the formation

of anion-associated complexes, highlighting increasing competition between water and anions for Li^+ coordination, consistent with the growth of anion–anion correlations and cation–anion antipeaks at high concentration.

5. Transport properties

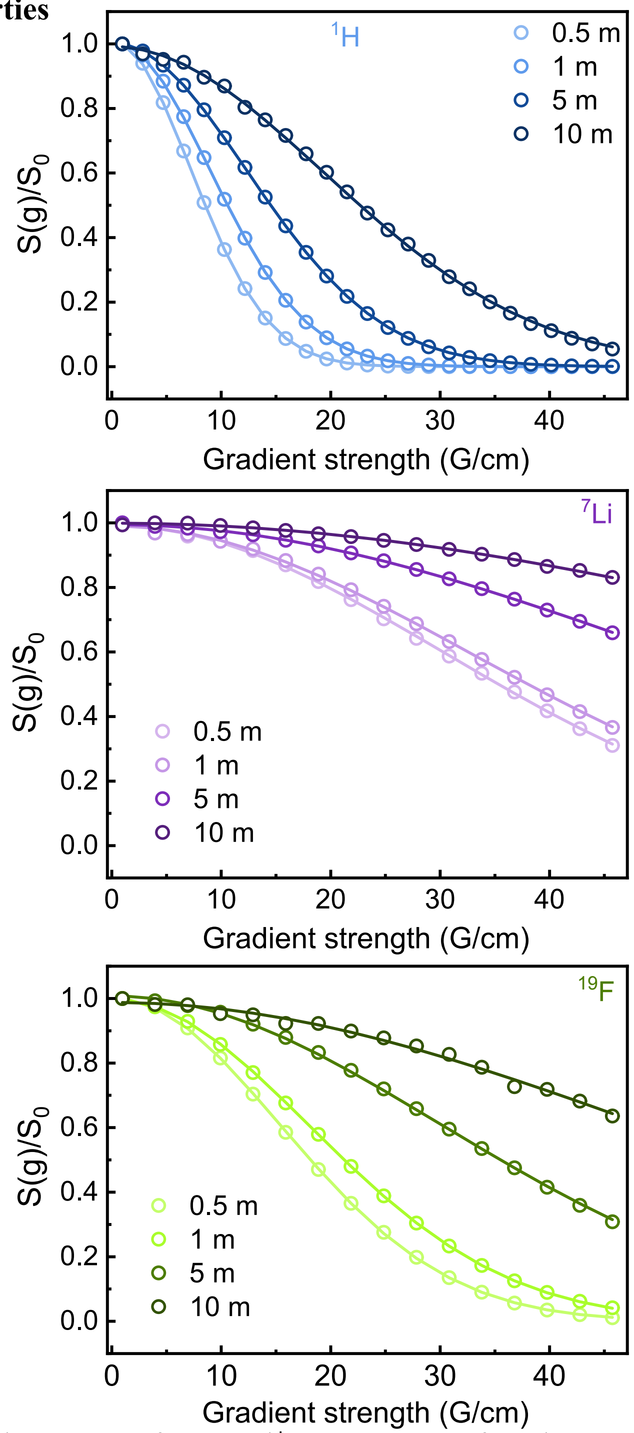

Figure S12. PFG fitting curve of H_2O, Li^+, BETI-BETI$^-$ for LiBETI-BETI aqueous solutions at different concentrations.

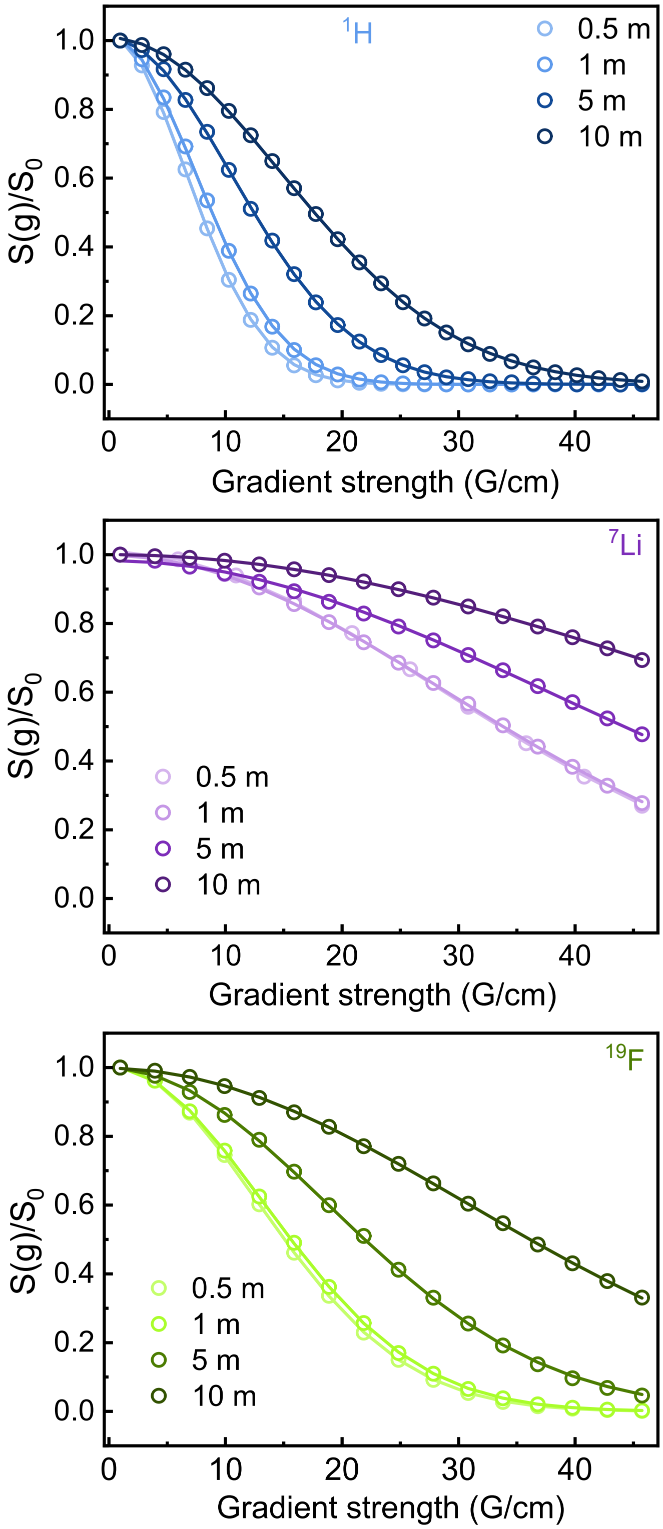

Figure S13. PFG fitting curve of H_2O, Li^+, BETI-FSI$^-$ for LiBETI-FSI aqueous solutions at different concentrations.

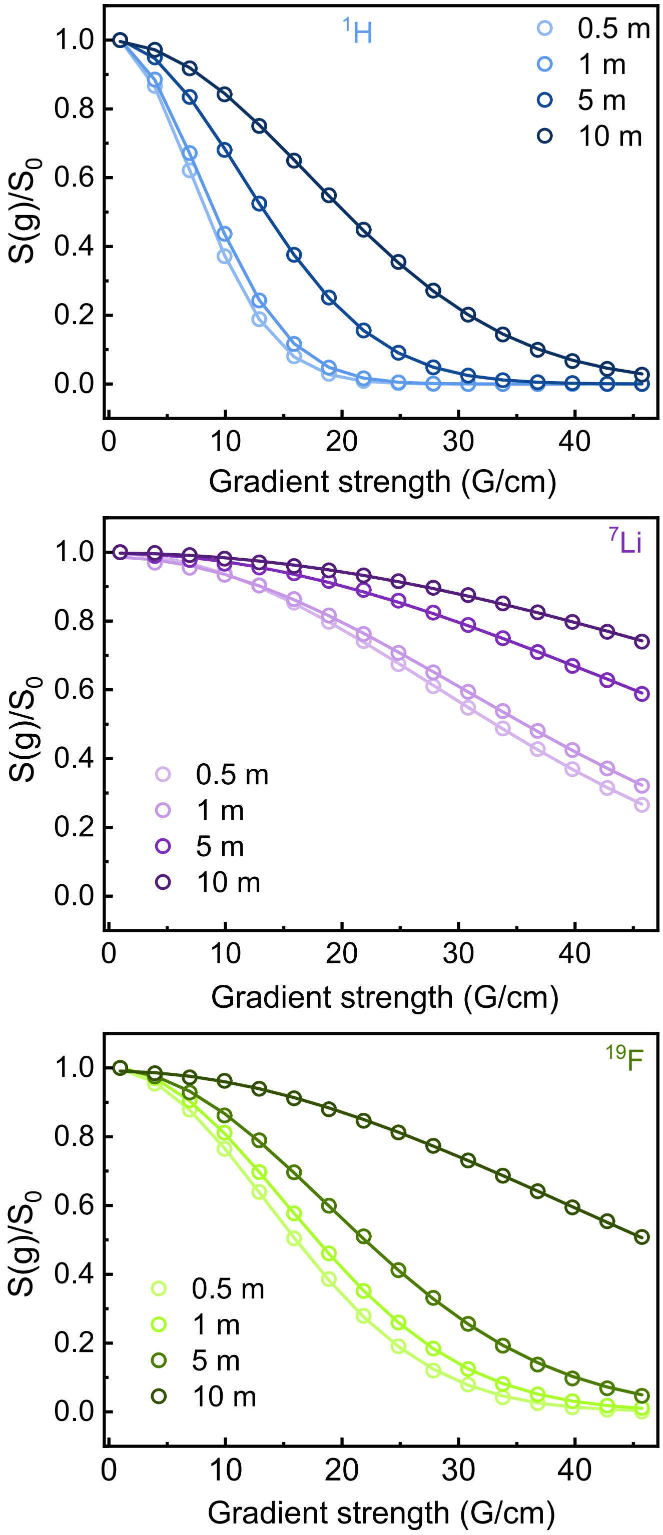

Figure S14. PFG fitting curve of H_2O, Li^+, BETI-TFSI$^-$ for LiBETI-TFSI aqueous solutions at different concentrations.

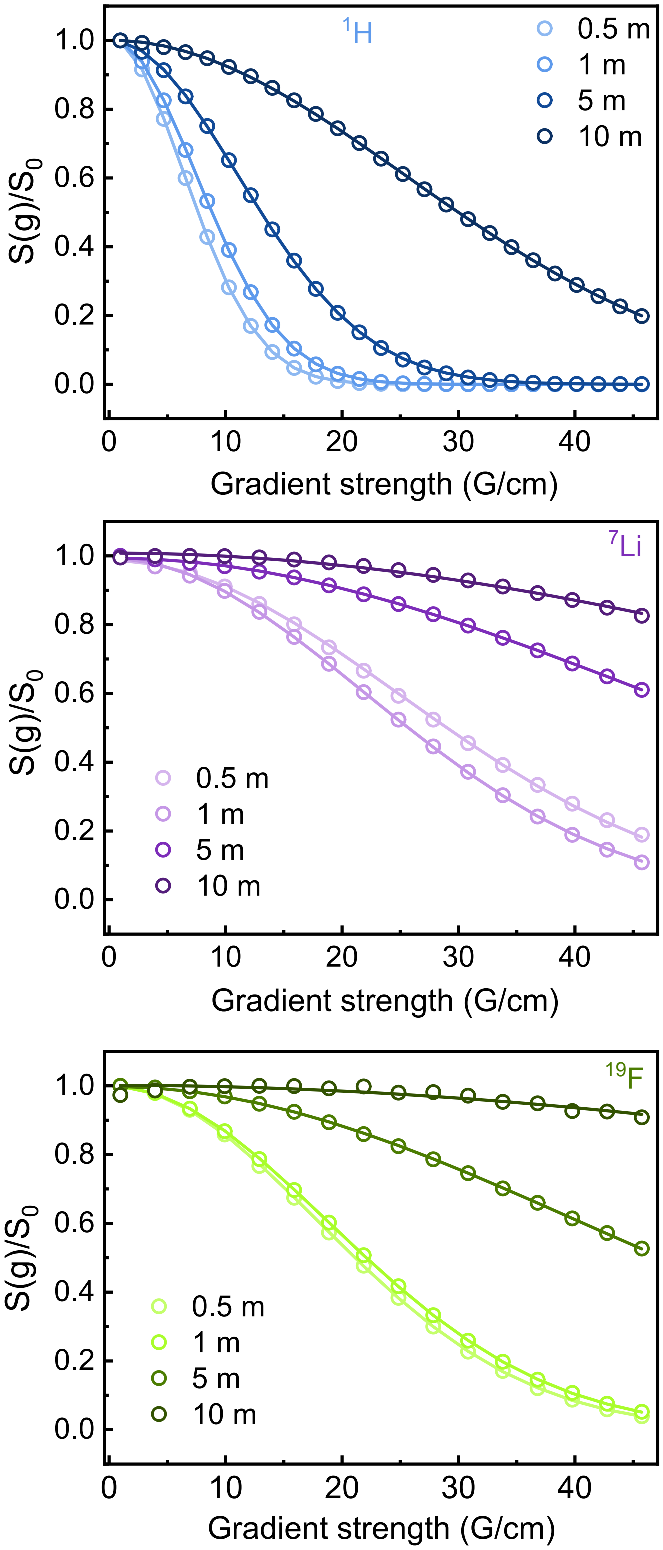

Figure S15. PFG fitting curve of H_2O, Li^+, BNTI-FSI $^-$ for LiBNTI-FSI aqueous solutions at different concentrations.

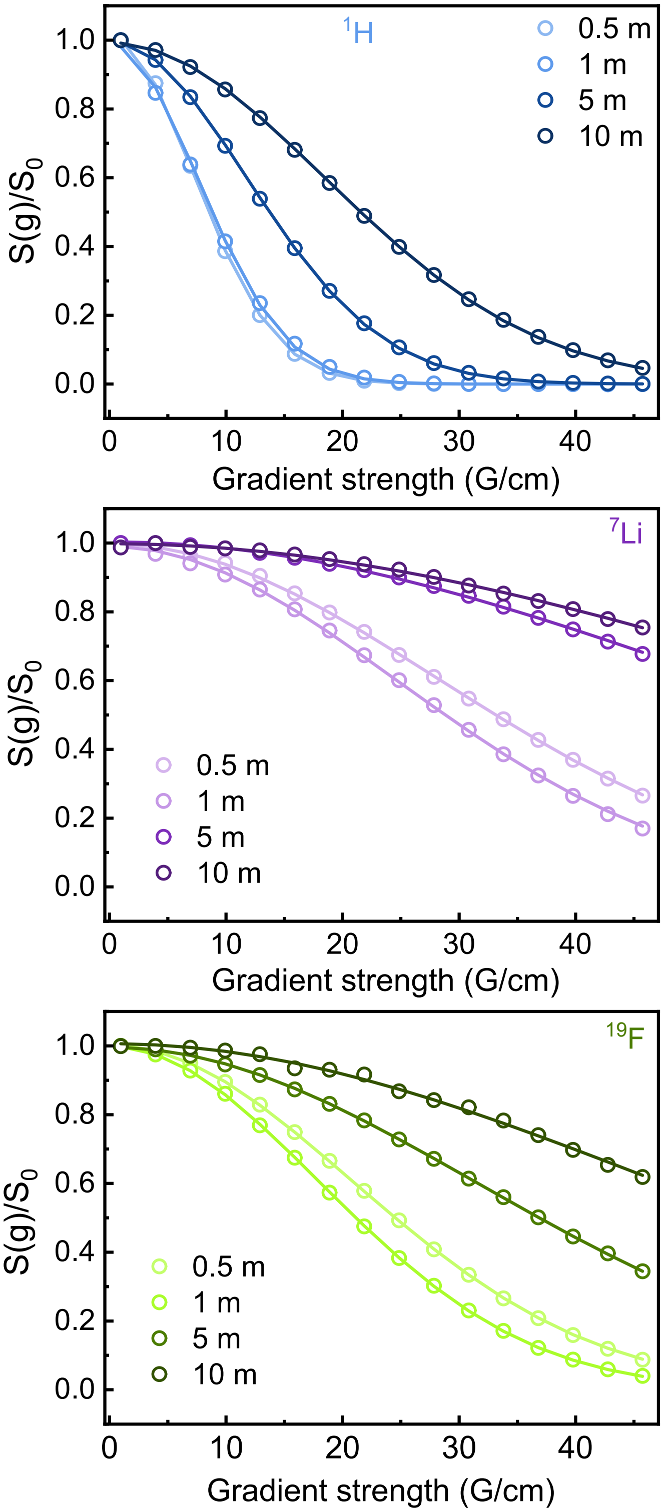

Figure S16. PFG fitting curve of H_2O, Li^+, BNTI-TFSI$^-$ for LiBNTI-TFSI aqueous solutions at different concentrations.

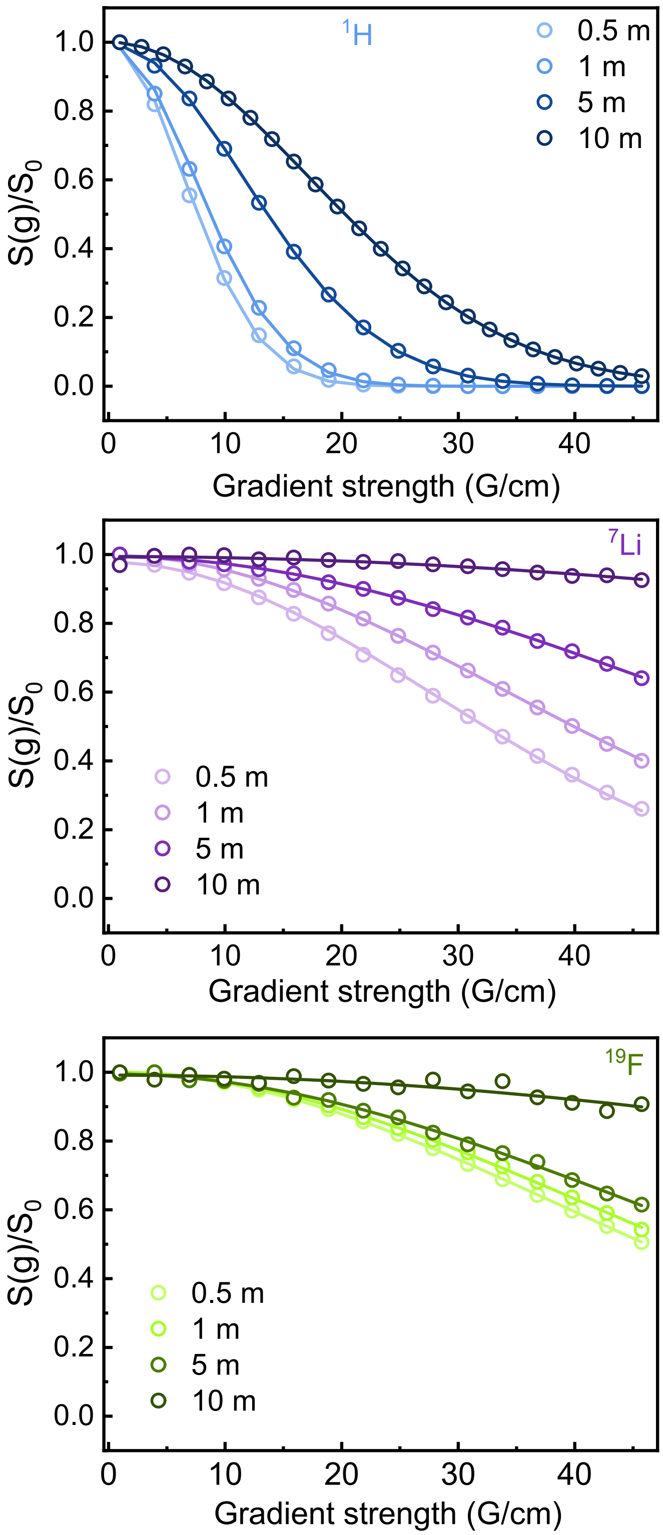

Figure S17. PFG fitting curve of H_2O, Li^+, $BNTI\text{-}BNTI^-$ for LiBNTI-BNTI aqueous solutions at different concentrations.

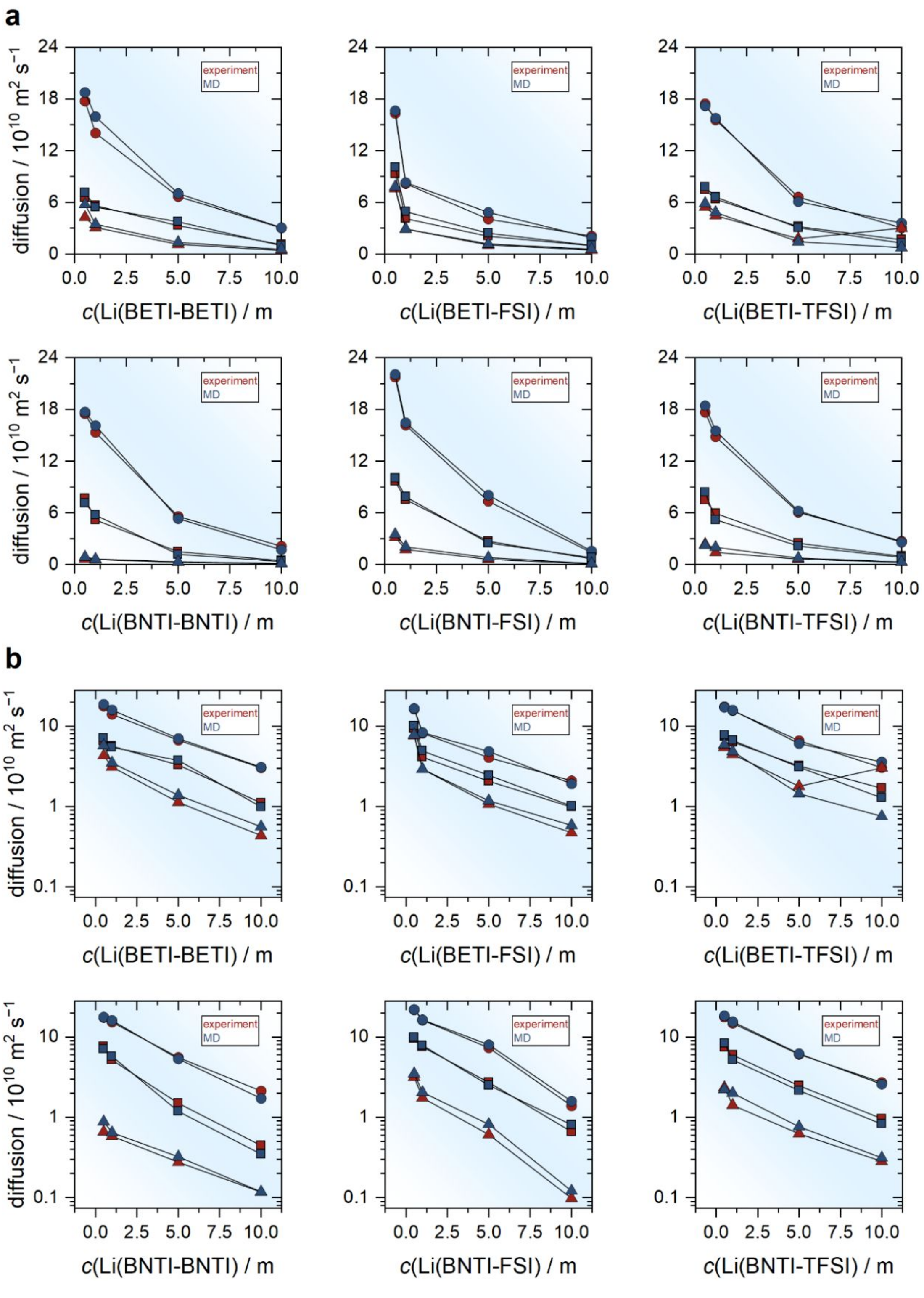

Figure S18. Diffusion coefficients of Li^+ (squares), anions (triangles), and H_2O (circles) at different concentrations for all Li imide-based salts aqueous solutions, comparing MD simulations (red) with experimental data (blue) in (a) linear and (b) logarithmic scale of *y*-axis.

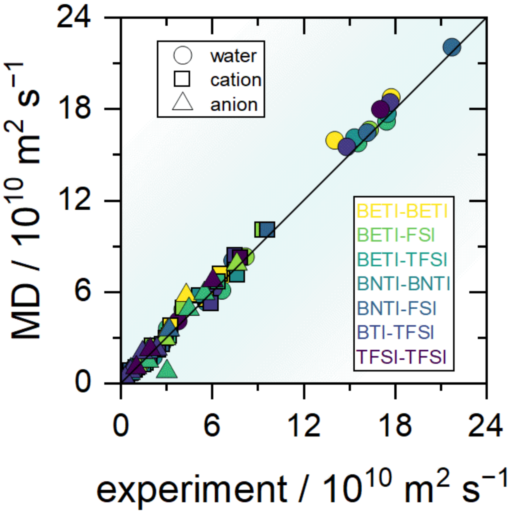

Figure S19. Comparing MD simulations with experimental data

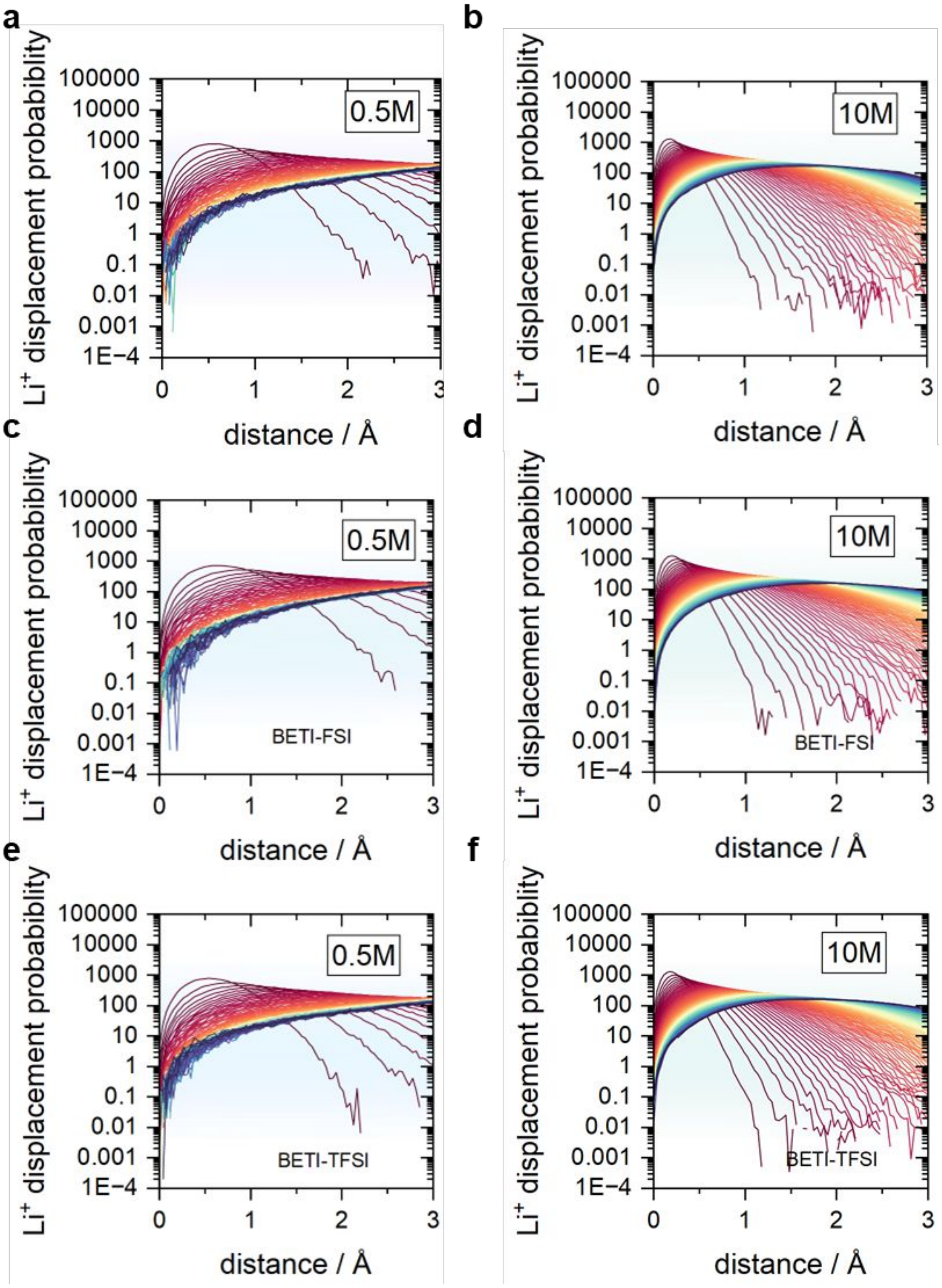

Figure S20. Van Hove probability distributions of Li^+ displacements at low (0.5 m) and high (10 m) concentrations in (a and b) LiBETI-BETI, (c and d) LiBETI-FSI, (e and f) LiBETI-TFSI aqueous electrolytes. The color gradient represents the evolution from short-time to long-time displacement probability distributions.
